# Supplementary material for: Platelets Increase the Expression of PD-L1 in Ovarian Cancer
Source: Cancers (Basel). 2022 May 19;14(10):2498. doi: 10.3390/cancers14102498 (PMC9139585; doi:10.3390/cancers14102498)
Supplement: Supplementary file 1 [file cancers-14-02498-s001.zip › cancers-1715797-suppl Figures.pdf]

Supplementary Data

Platelets Increase the Expression of PD-L1 in Ovarian Cancer

Min Soon Cho[1*], Hani Lee[1#], Ricardo Gonzalez-Delgado[1#], Dan Li[2], Tomoyuki Sasano[3], Wendolyn Carlos-Alcalde[1], Qing Ma[2], Jinsong Liu[4], Anil K. Sood[3], Vahid Afshar-Kharghan[1]

[1]Section of Benign Hematology, University of Texas MD Anderson Cancer Center, Houston, Texas, USA

[2]Department of Hematopoietic Biology and Malignancy, University of Texas MD Anderson Cancer Center, Houston, Texas, USA

[3]Department of Gynecologic Oncology and Reproductive Medicine, University of Texas MD Anderson Cancer Center, Houston, Texas, USA

[4]Department of Pathology, University of Texas MD Anderson Cancer Center, Houston, Texas, USA

Running title: Platelets promote the expression of PD-L1 in ovarian cancer.

* Correspondence:mscho@mdanderson.org

Min Soon Cho, PhD

[1]Section of Benign Hematology

MD Anderson Cancer Center

Z9.5042

6565 MD Anderson Blvd.

Houston, Texas 77030, USA

# Contributed equally to the manuscript

SUPPLEMENTARY LEGENDS AND FIGURES

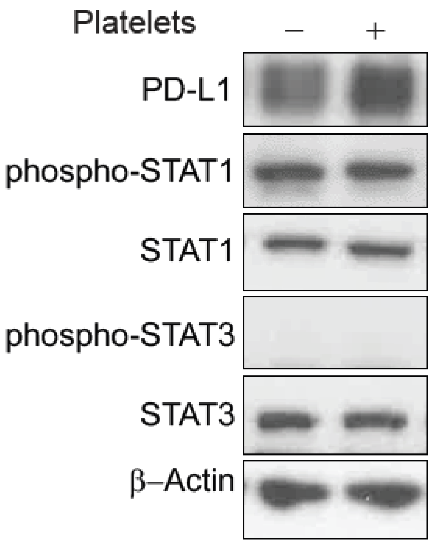

Figure S1. Platelet-enhanced PD-L1 expression in ovarian cancer cells is independent of STAT1/3 phosphorylation. Whole-cell lysates from OVCAR8 ovarian cancer cells incubated with or without platelets were immunoblotted for PD-L1 and phosphorylated STAT1 and STAT3. Protein bands representing β–Actin, total STAT1, and STAT3 served as loading controls. The data is representative of three independent experiments.

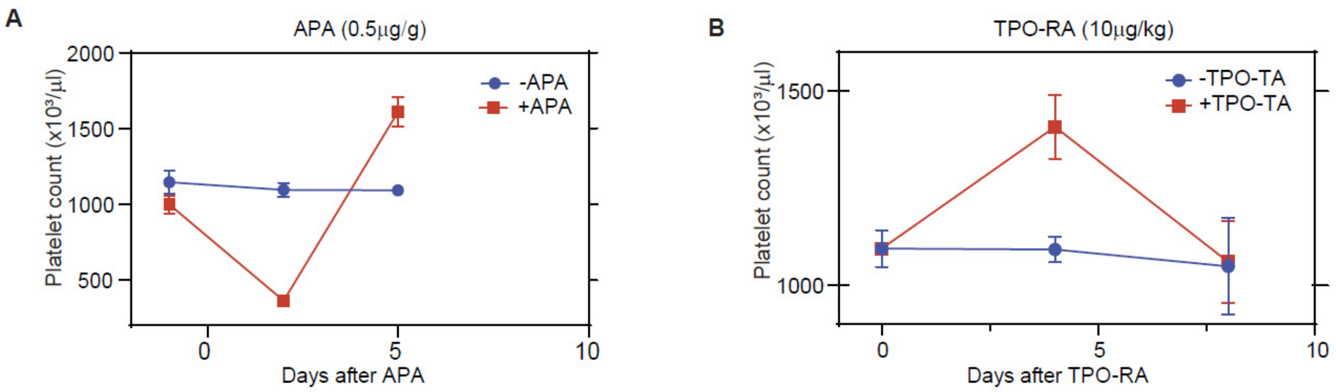

Figure S2. Effect of antiplatelet antibody (APA) and murine thrombopoietin receptor agonist (TPO-RA) on platelet counts in mice. We measured platelet counts at regular intervals in mice treated with (**A**) APA (0.5µg/g) or **(B)** TPO-RA (10 µg/kg).

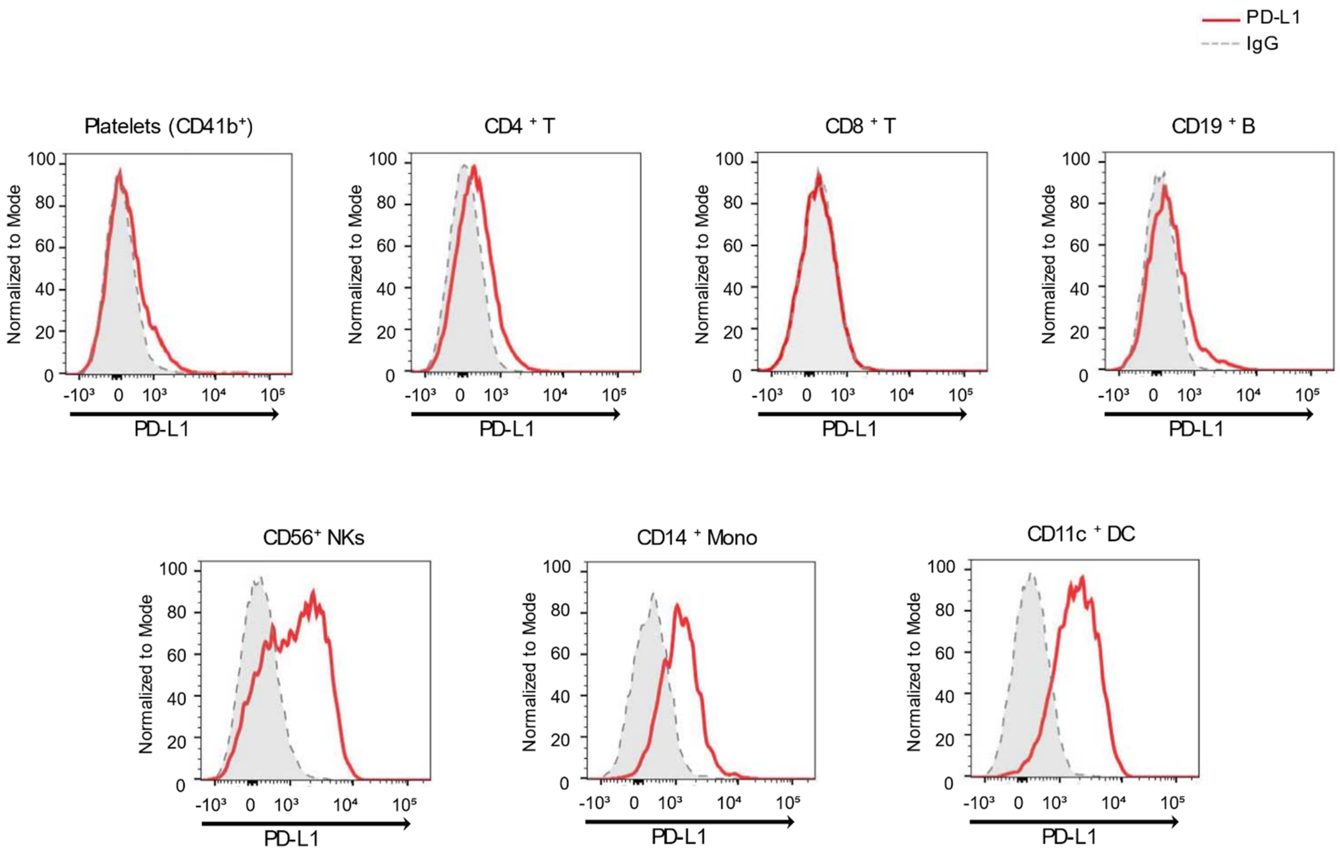

Figure S3. Expression of PD-L1on peripheral blood cells from a healthy donor. We used flow cytometry to investigate the expression of PD-L1 on peripheral blood cells. We compared the binding of the anti-PDL-1 antibody to that of control IgG to CD41b+ Platelets, CD4+ T cells, CD8+ T cells, CD19+ B cells, CD56+ Natural-Killer (NK) cells, CD14+ monocytes (Mono), and CD11c+ Dendritic cells (DC).

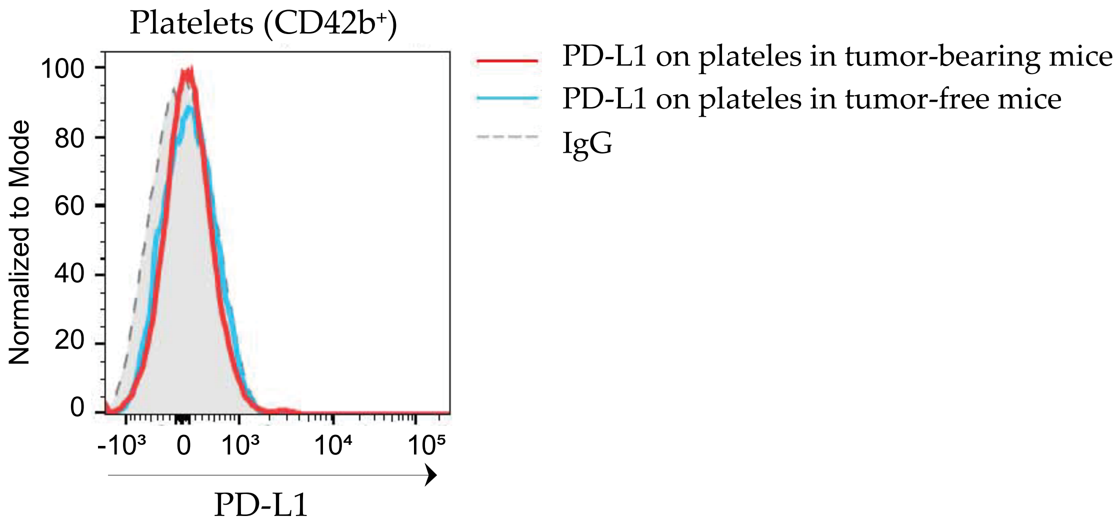

Figure S4. Expression of PD-L1 on platelets in tumor-bearing and tumor-free mice. We used flow cytometry to compare the binding of anti-PD-L1 or control IgG to CD42b+ platelets from ovarian tumor-bearing mice or tumor-free mice.

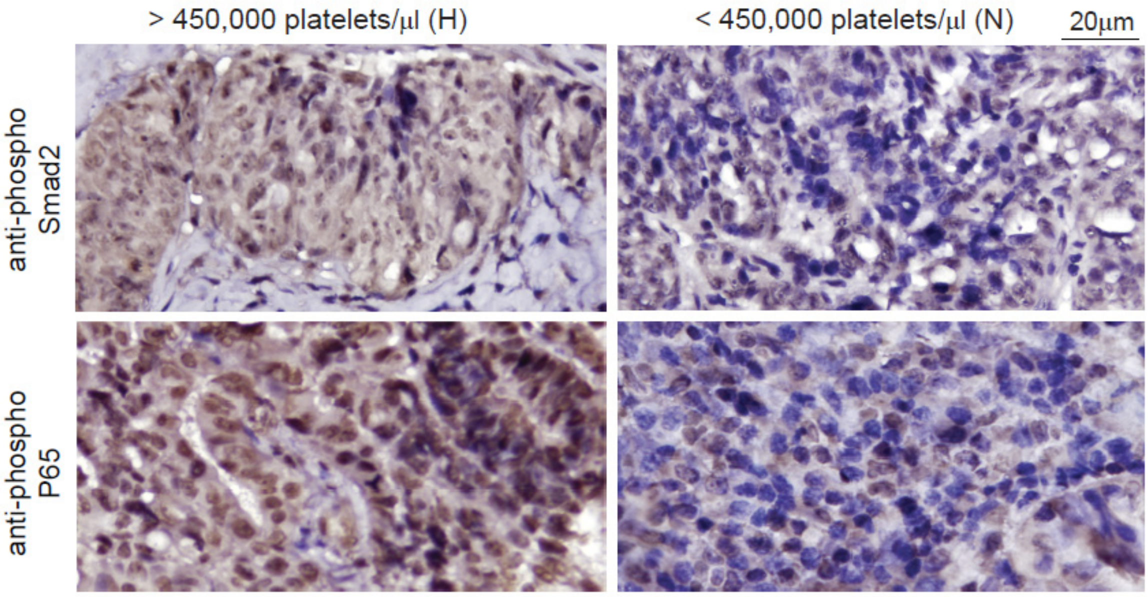

Figure S5. Increase in the expression of Phospho-Smad2 and phospho-p65 expression in ovarian cancer tissue specimens in the presence of thrombocytosis. We stained tumor specimens from patients with elevated and normal platelet counts with antibodies against phospho-Smad2 or phospho-p65.
