# Supplementary material for: Platelets Increase the Expression of PD-L1 in Ovarian Cancer
Source: Cancers (Basel). 2022 May 19;14(10):2498. doi: 10.3390/cancers14102498 (PMC9139585; doi:10.3390/cancers14102498)

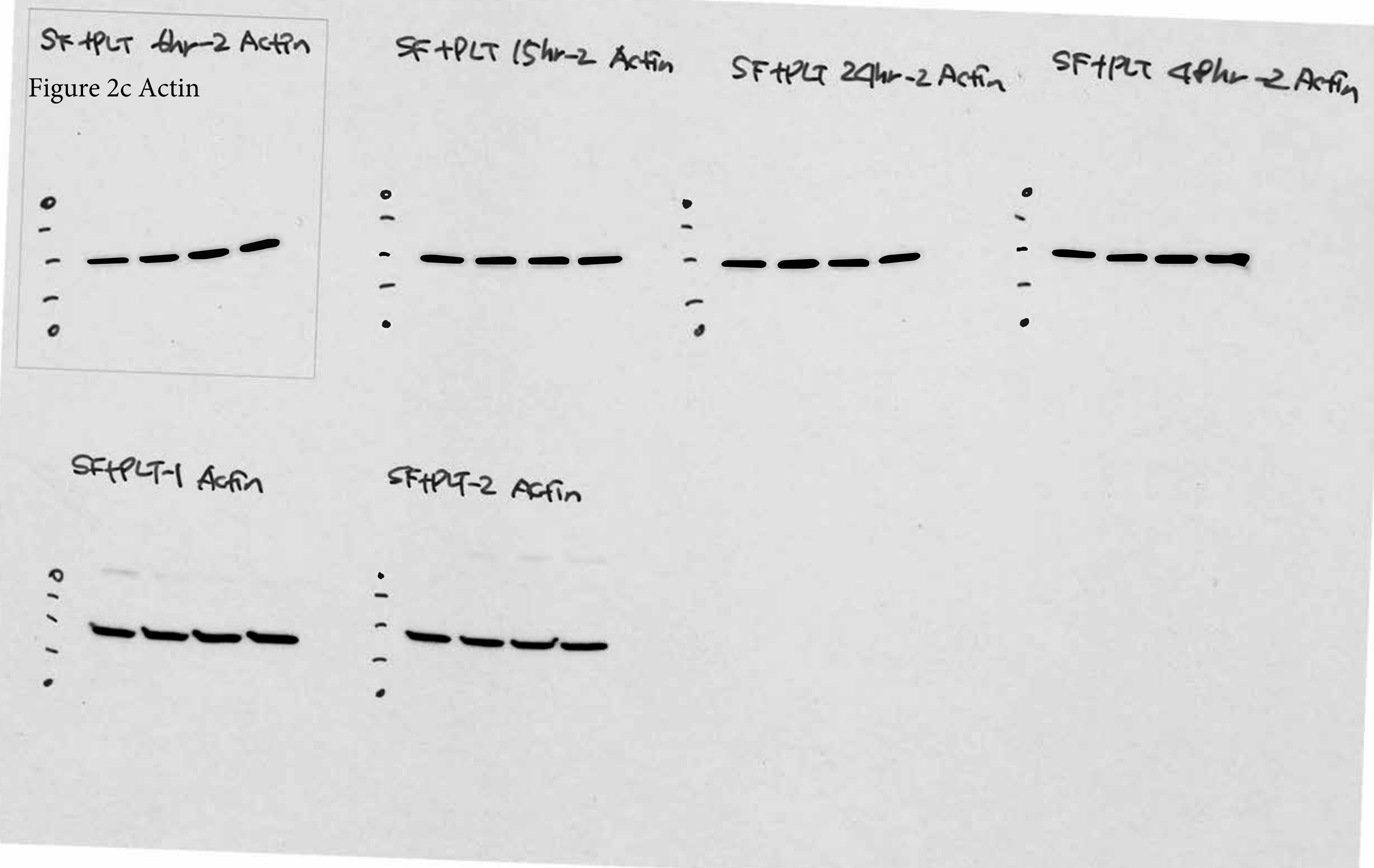

Figure 2c Actin

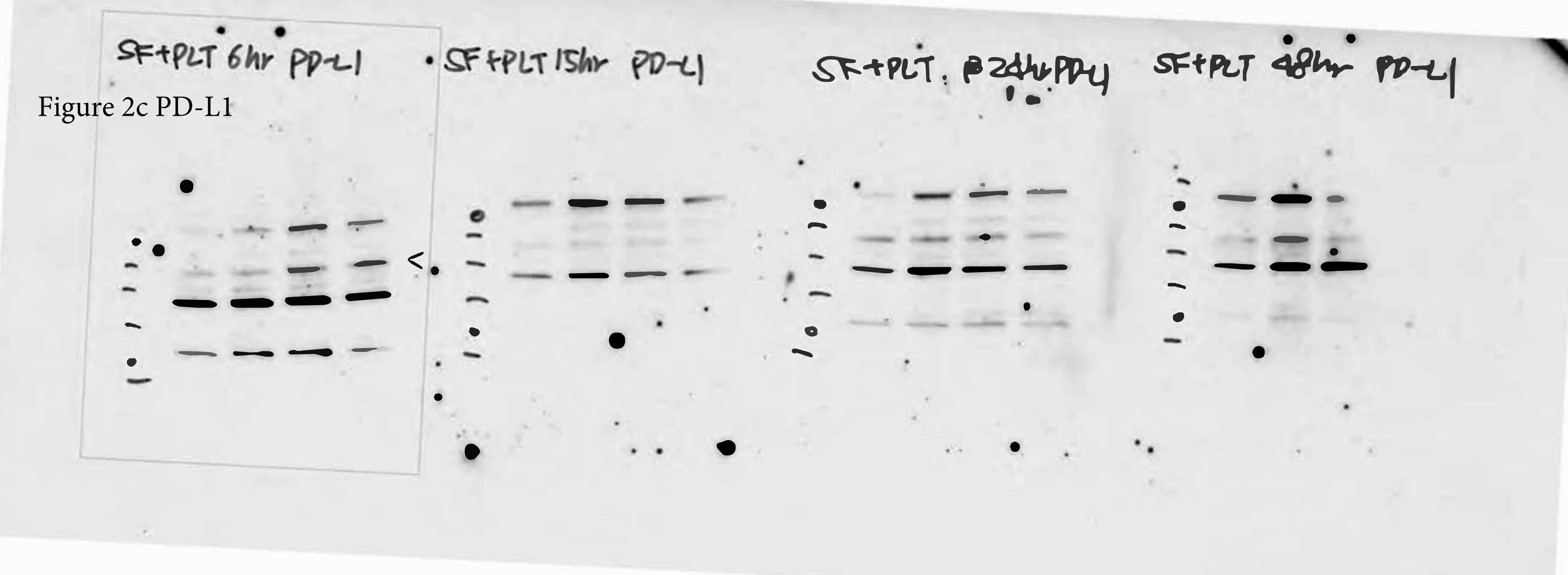

Figure 2c PD-L1

Figure 3a Actin

Fig3a Actin

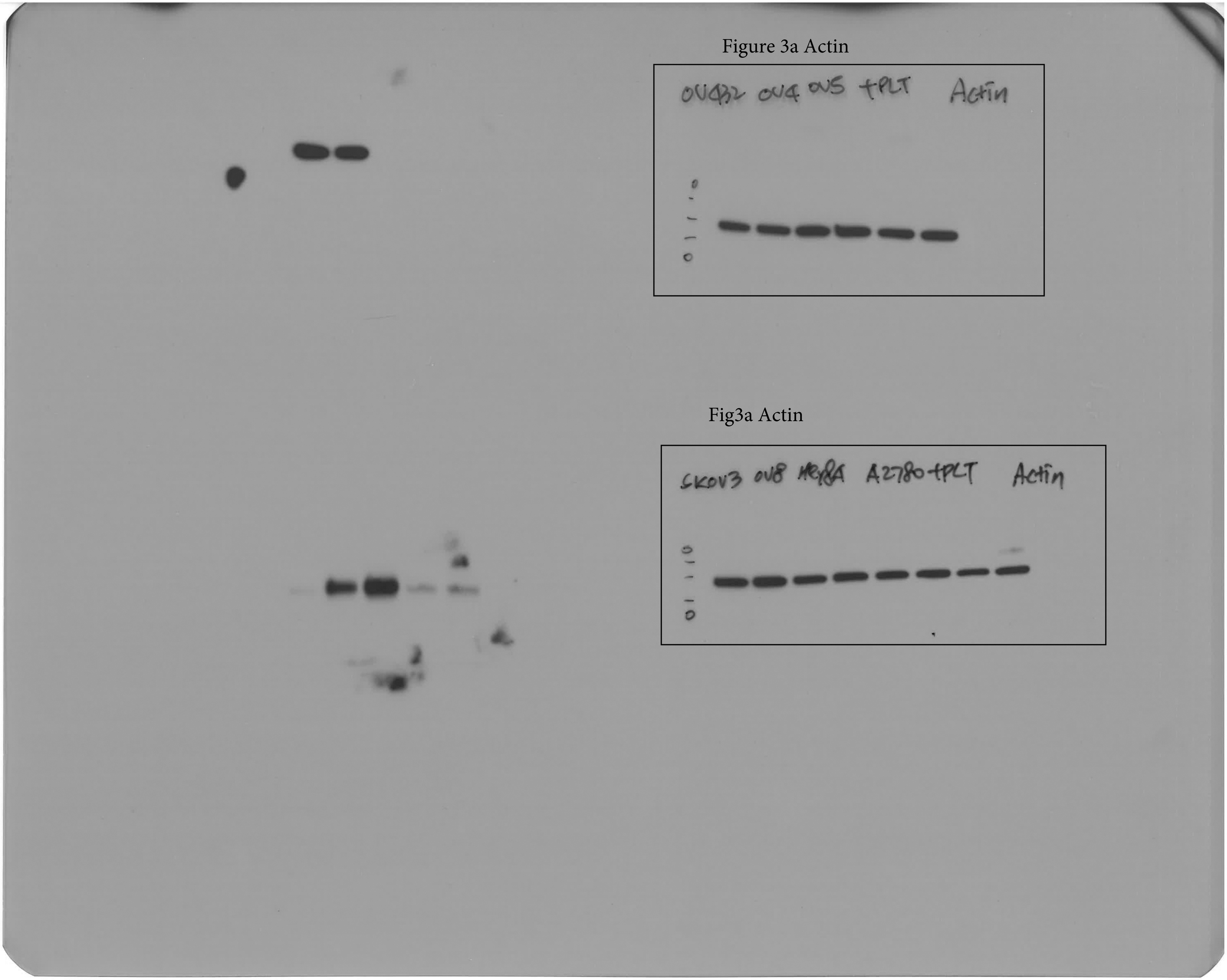

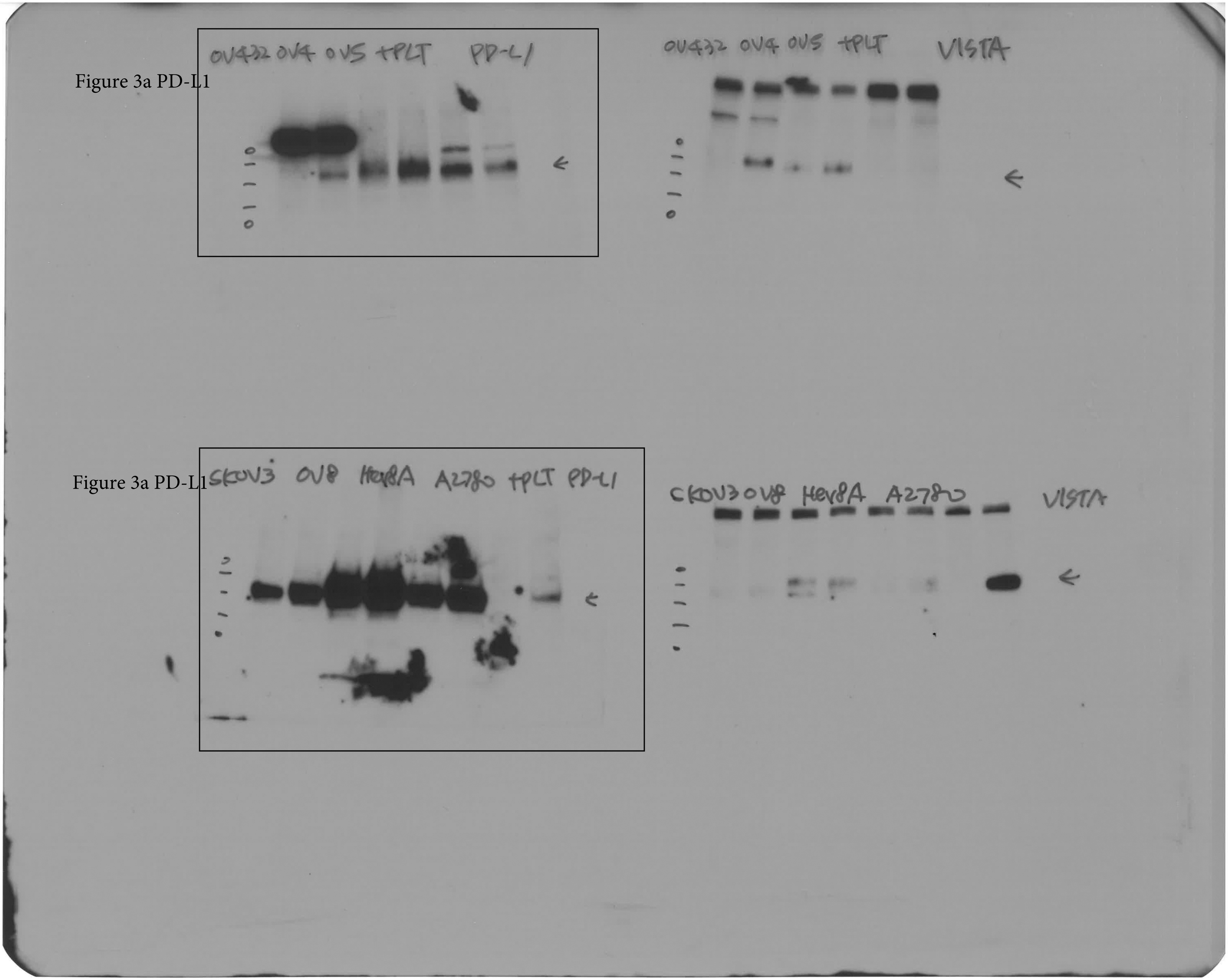

Figure 3a PD-L1

Figure 3a PD-L1

Figure 3b GAPDH

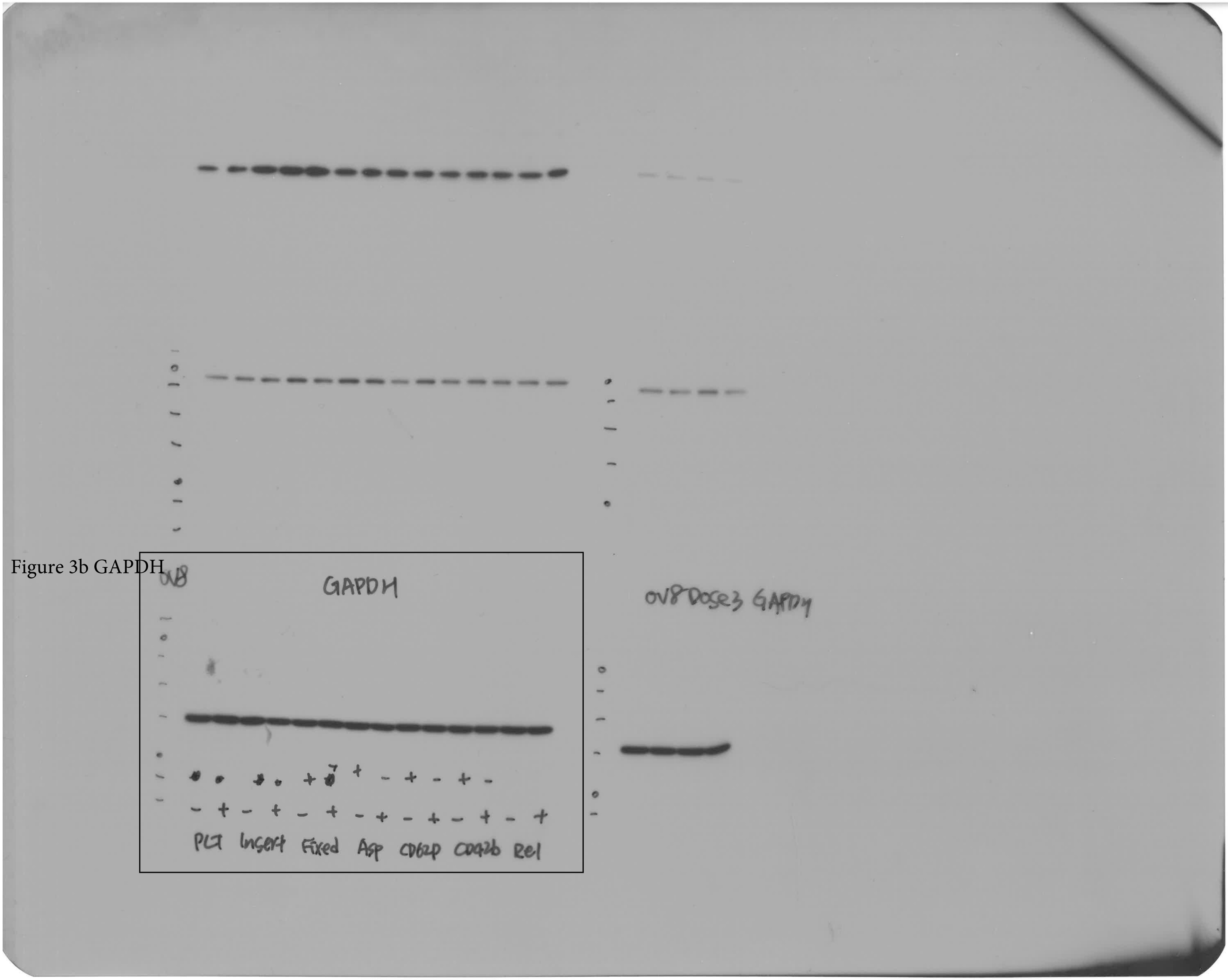

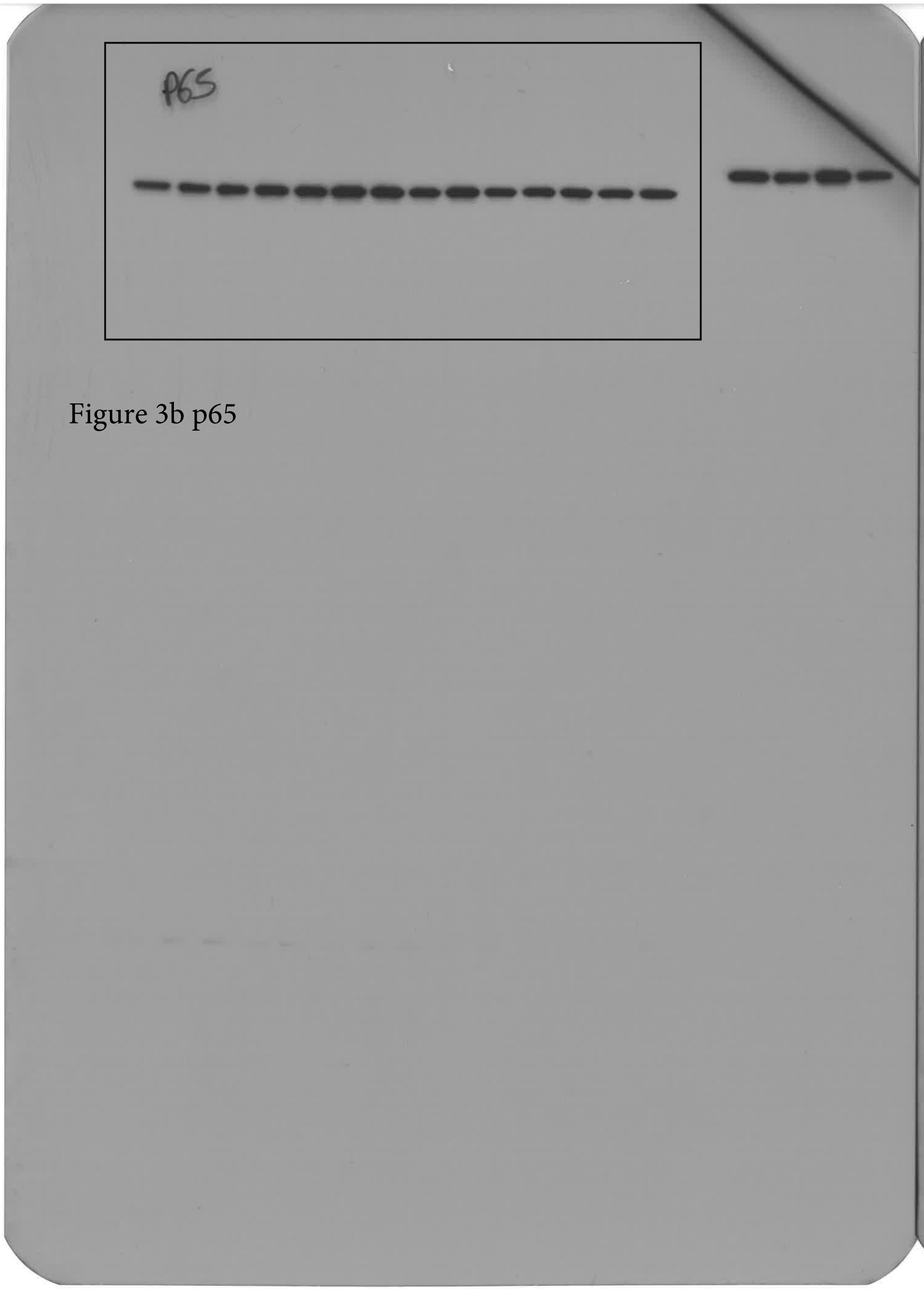

Figure 3b p65

Figure 3b Smad2/3

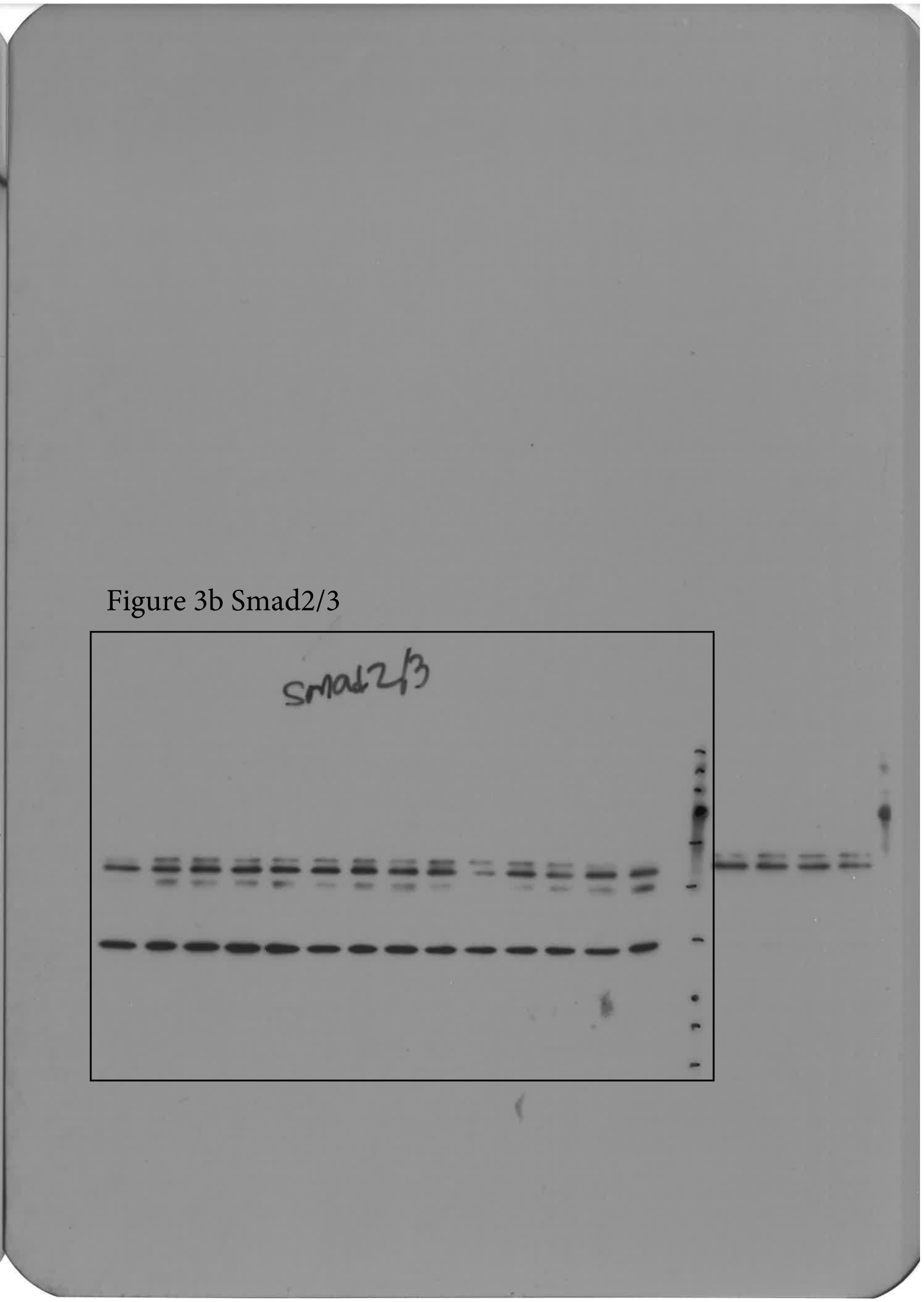

Figure 3b PD-L1

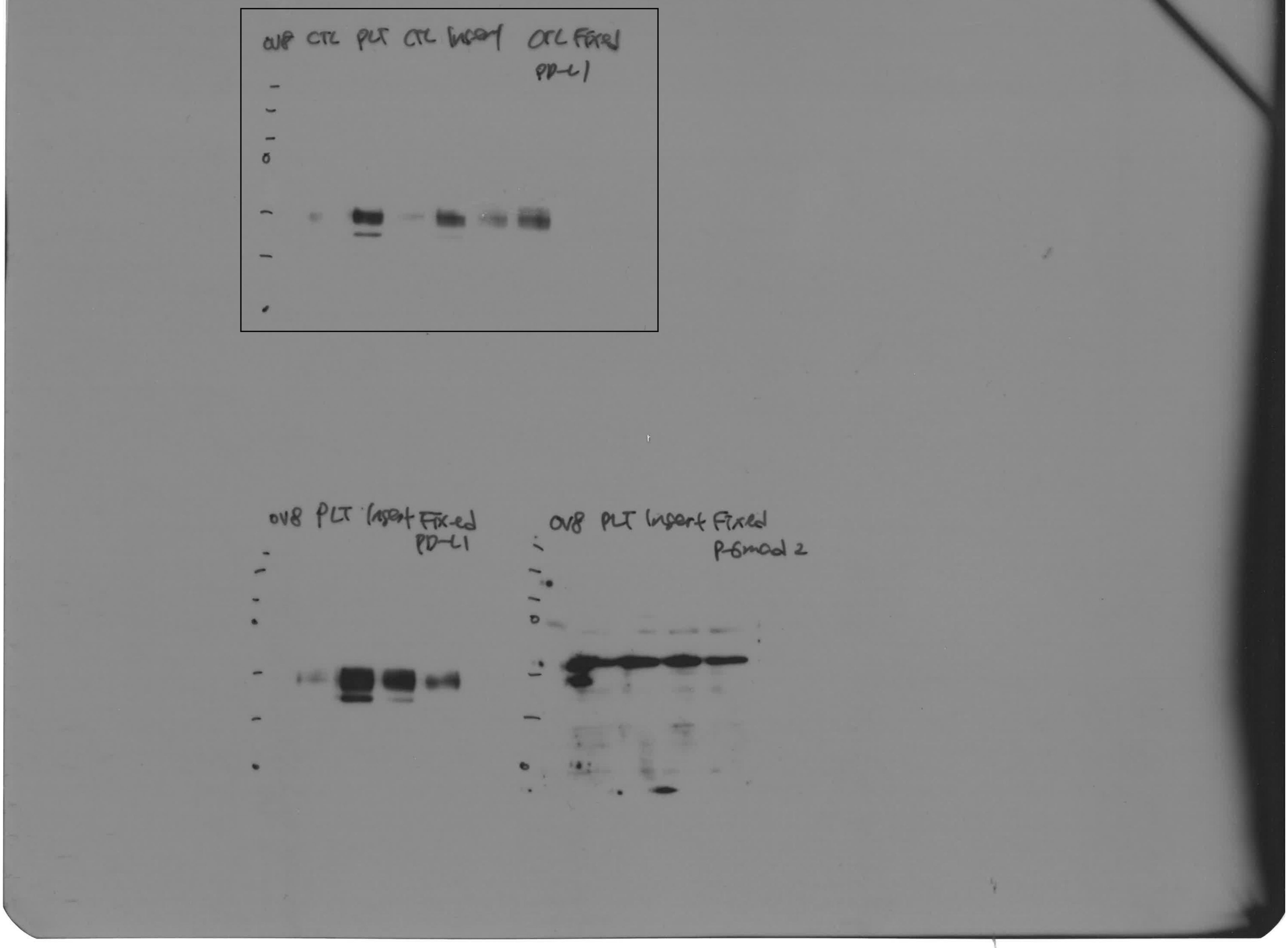

Figure 3b pP65

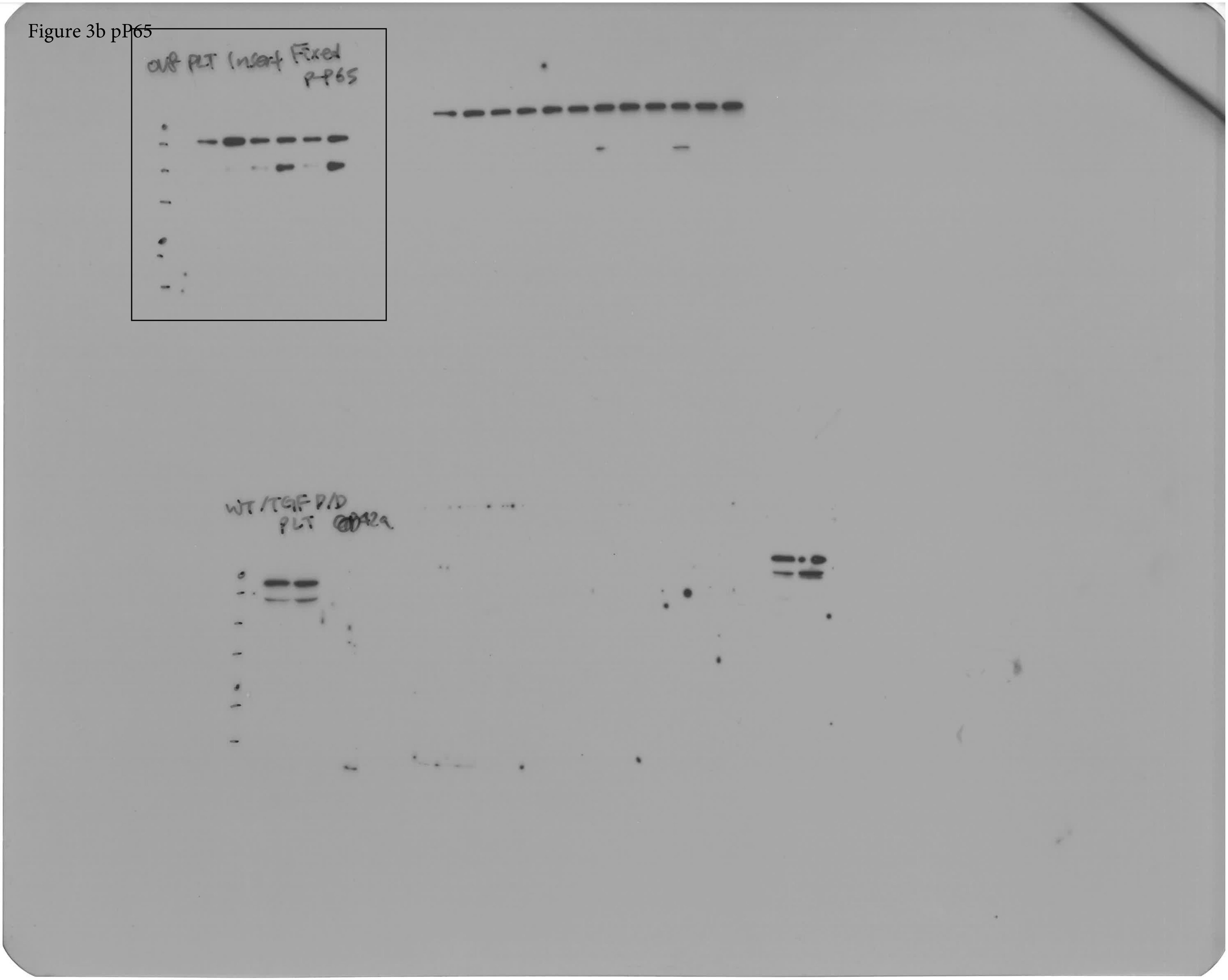

Figure 3b pSmad2

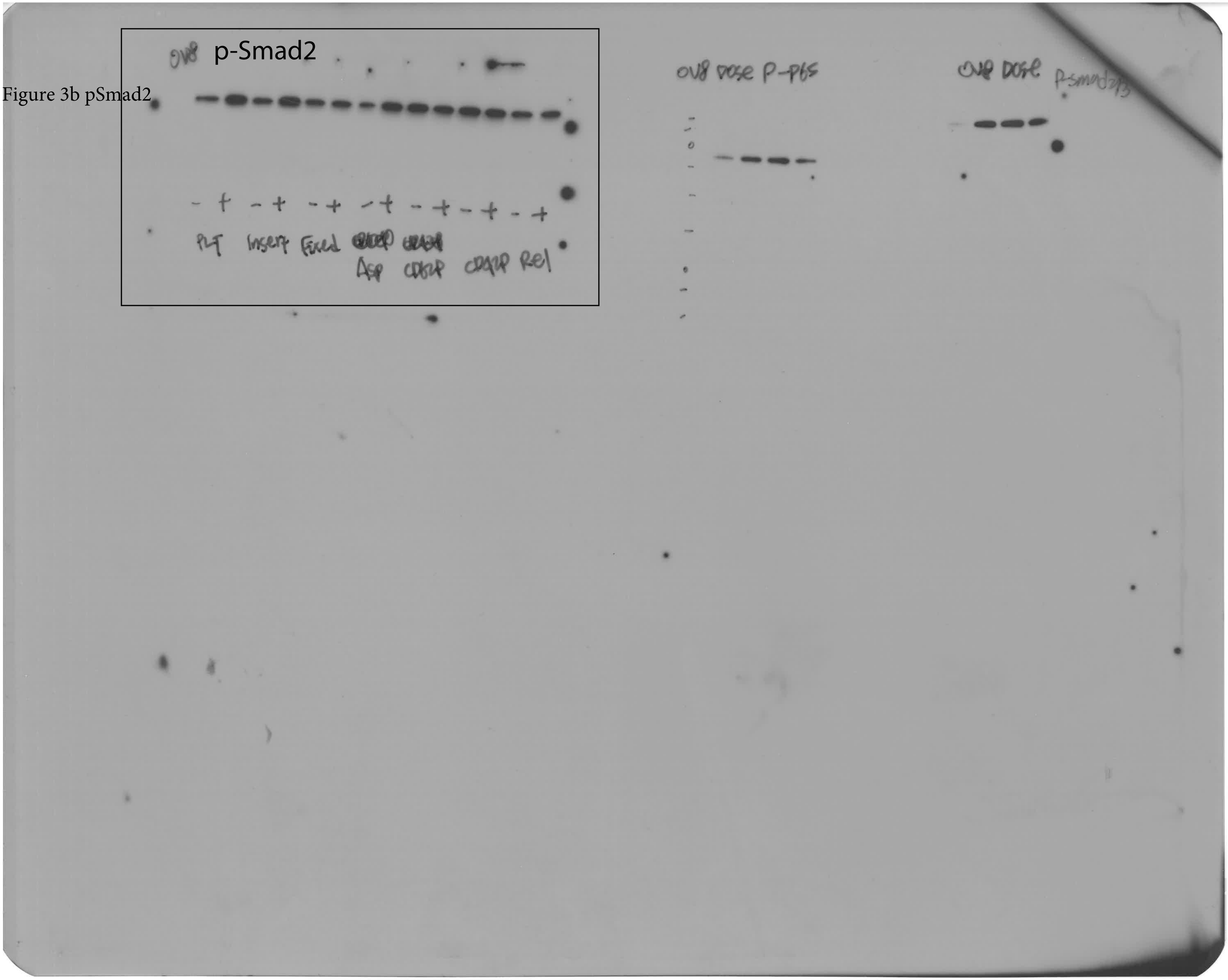

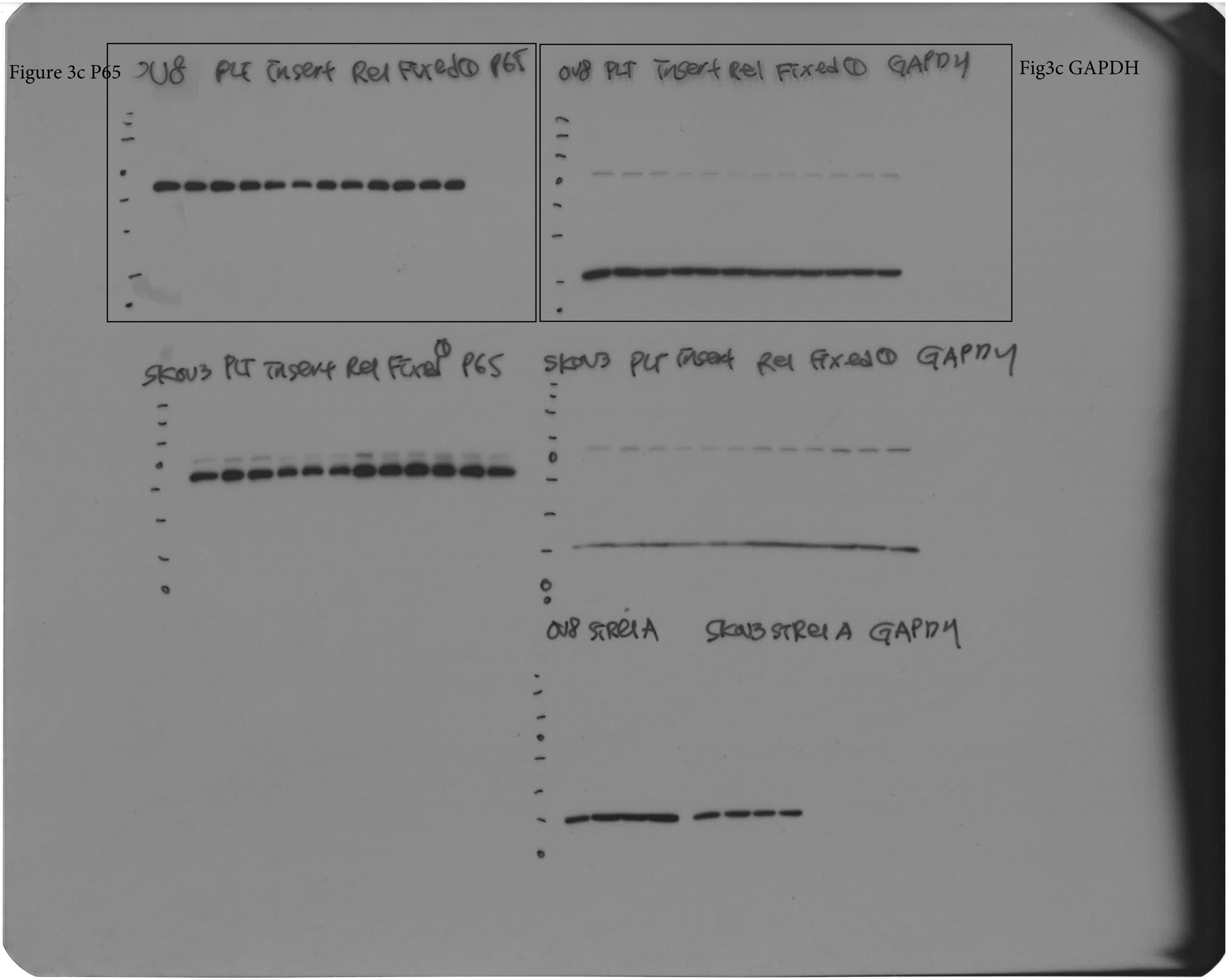
Figure 3c P65
OV8 PLT Insert Rel Fixed① P65
OV8 PLT Insert Rel Fixed① GAPDH
Fig3c GAPDH
SKOV3 PLT Insert Rel Fixed① P65
SKOV3 PLT Insert Rel Fixed① GAPDH
OV8 siRelA SKOV3 siRelA GAPDH

Figure 3c PD-L1

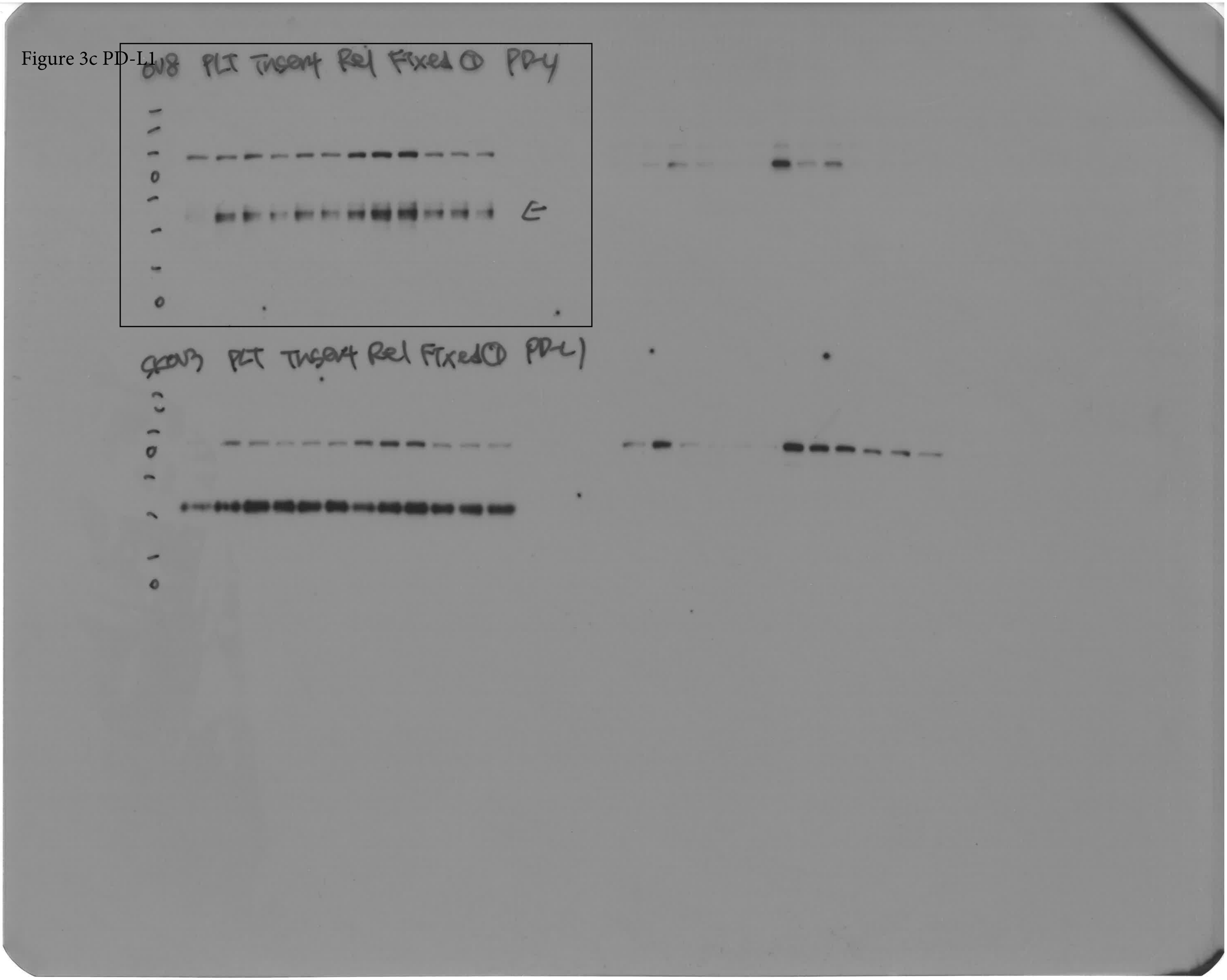

Figure 3c pP65

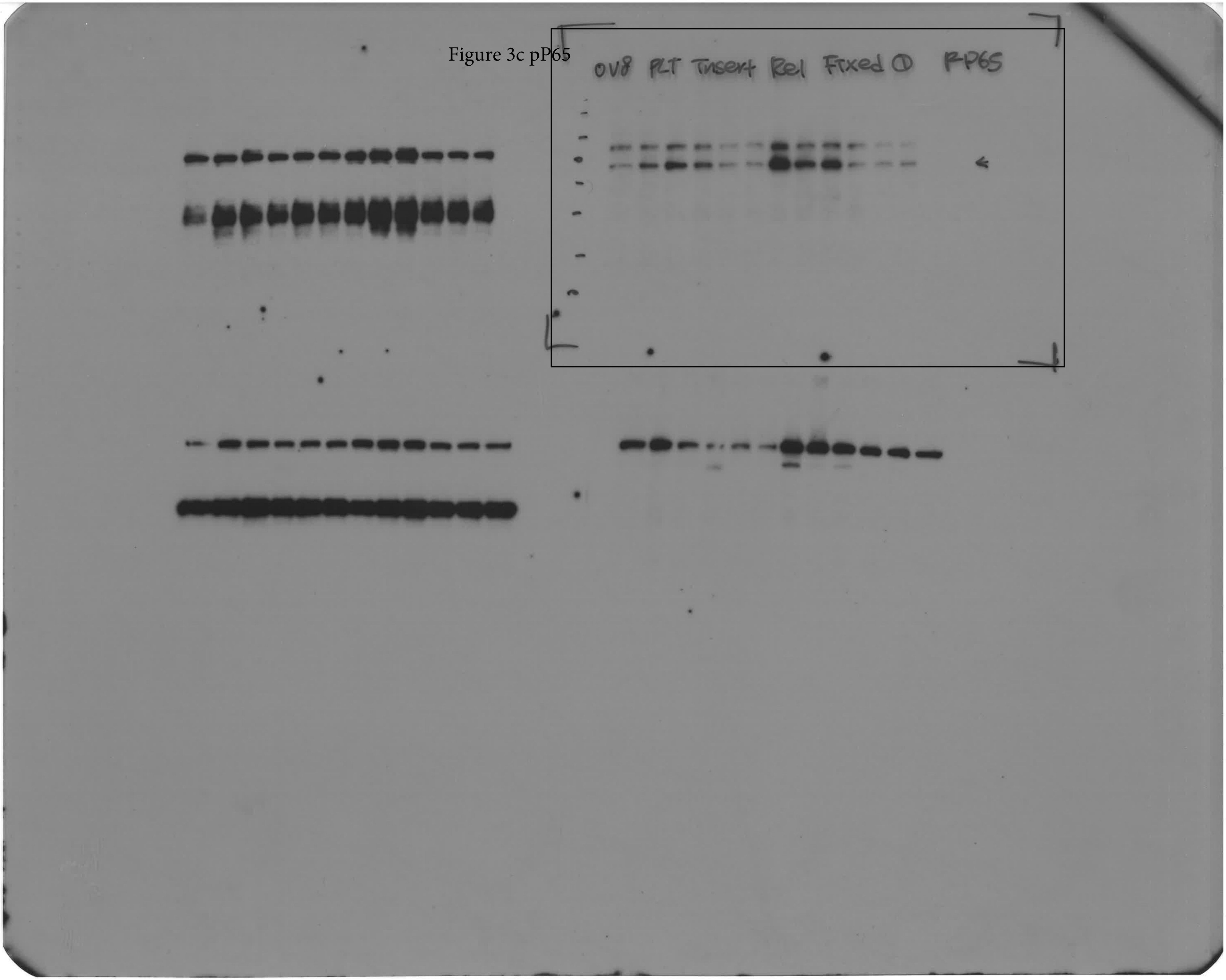

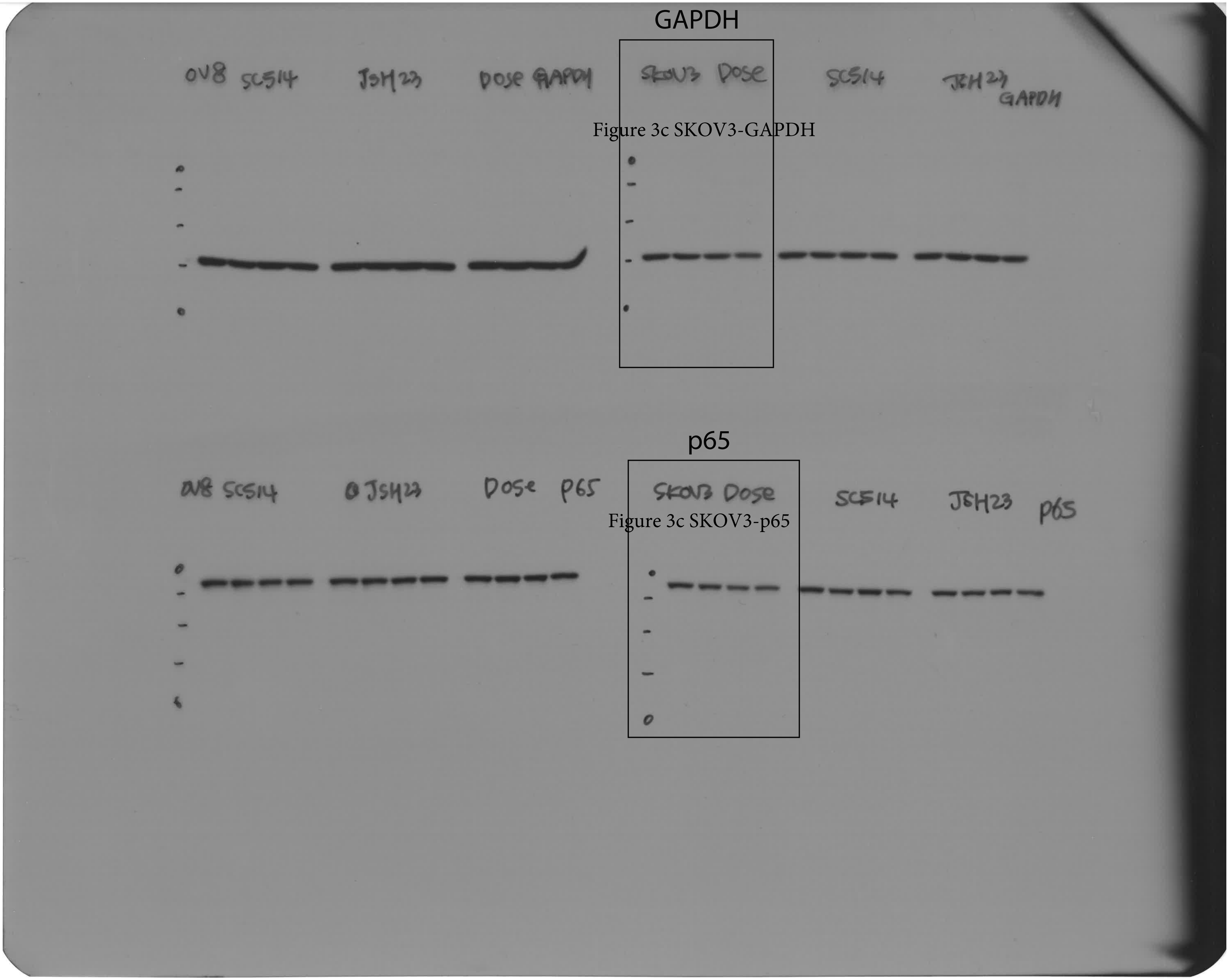
GAPDH
OV8 SC514 JSH23 Dose GAPDH
SKOV3 Dose SC514 JSH23 GAPDH
Figure 3c SKOV3-GAPDH
p65
OV8 SC514 JSH23 Dose p65
SKOV3 Dose SC514 JSH23 p65
Figure 3c SKOV3-p65

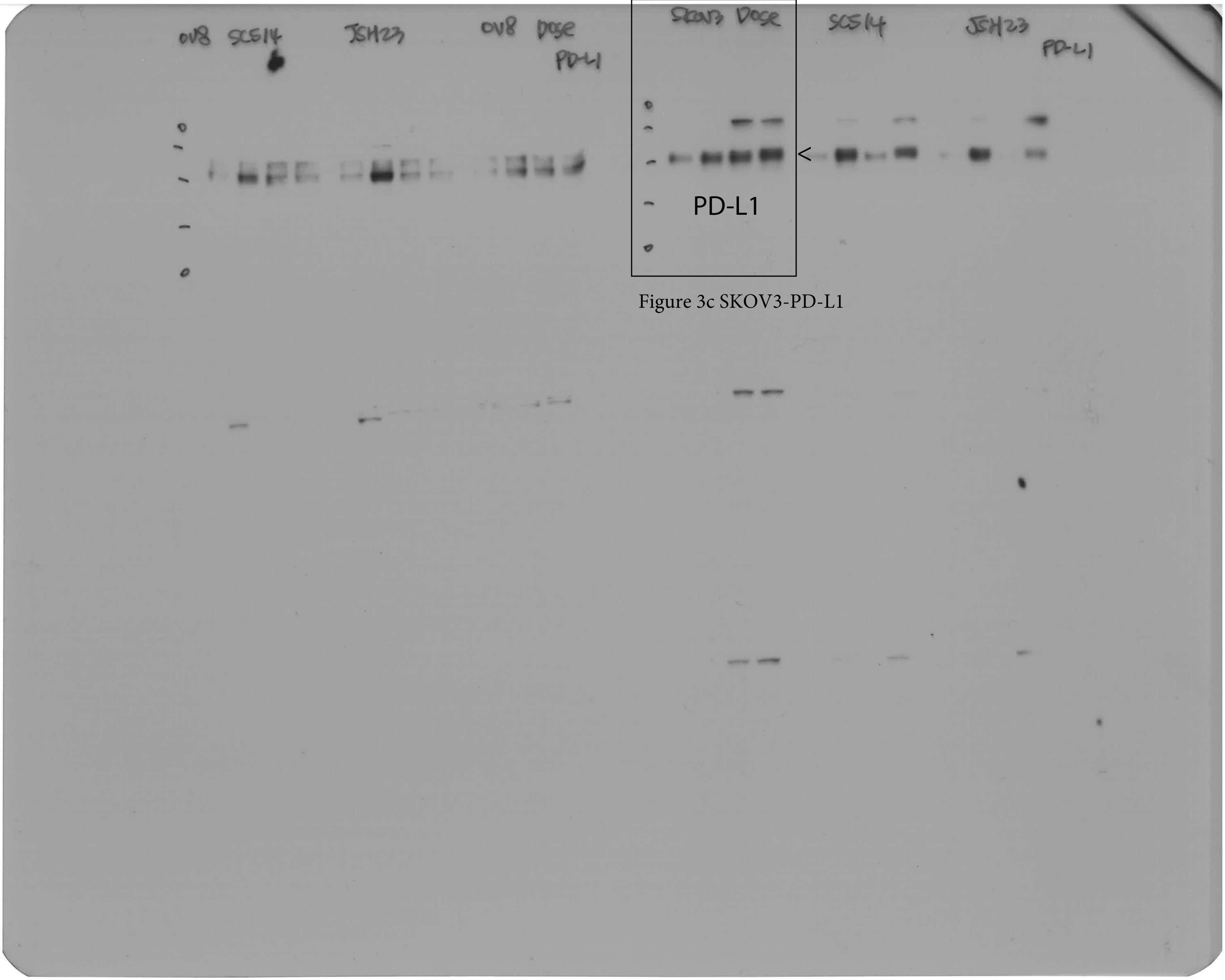

Figure 3c SKOV3-PD-L1

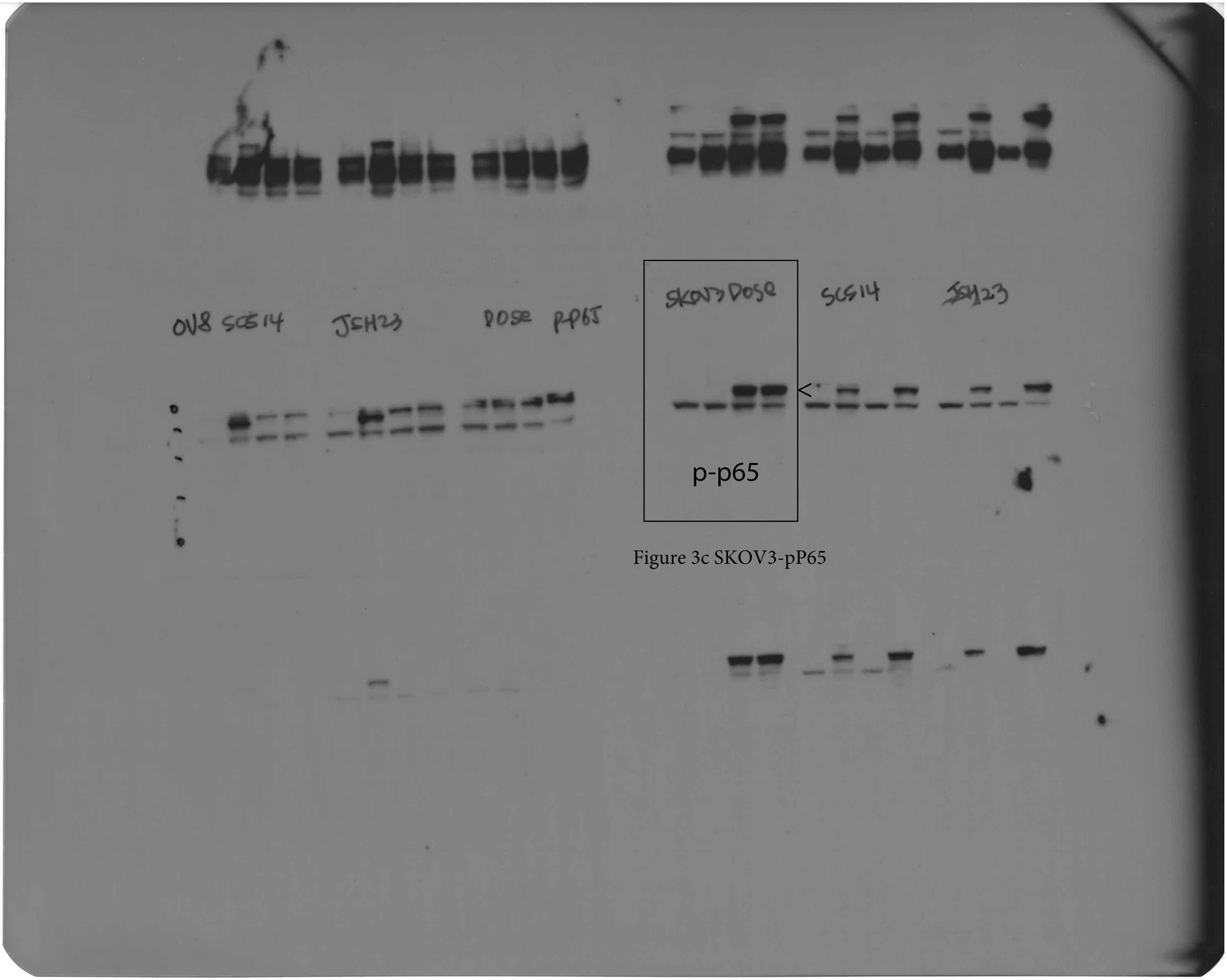

Figure 3c SKOV3-pP65

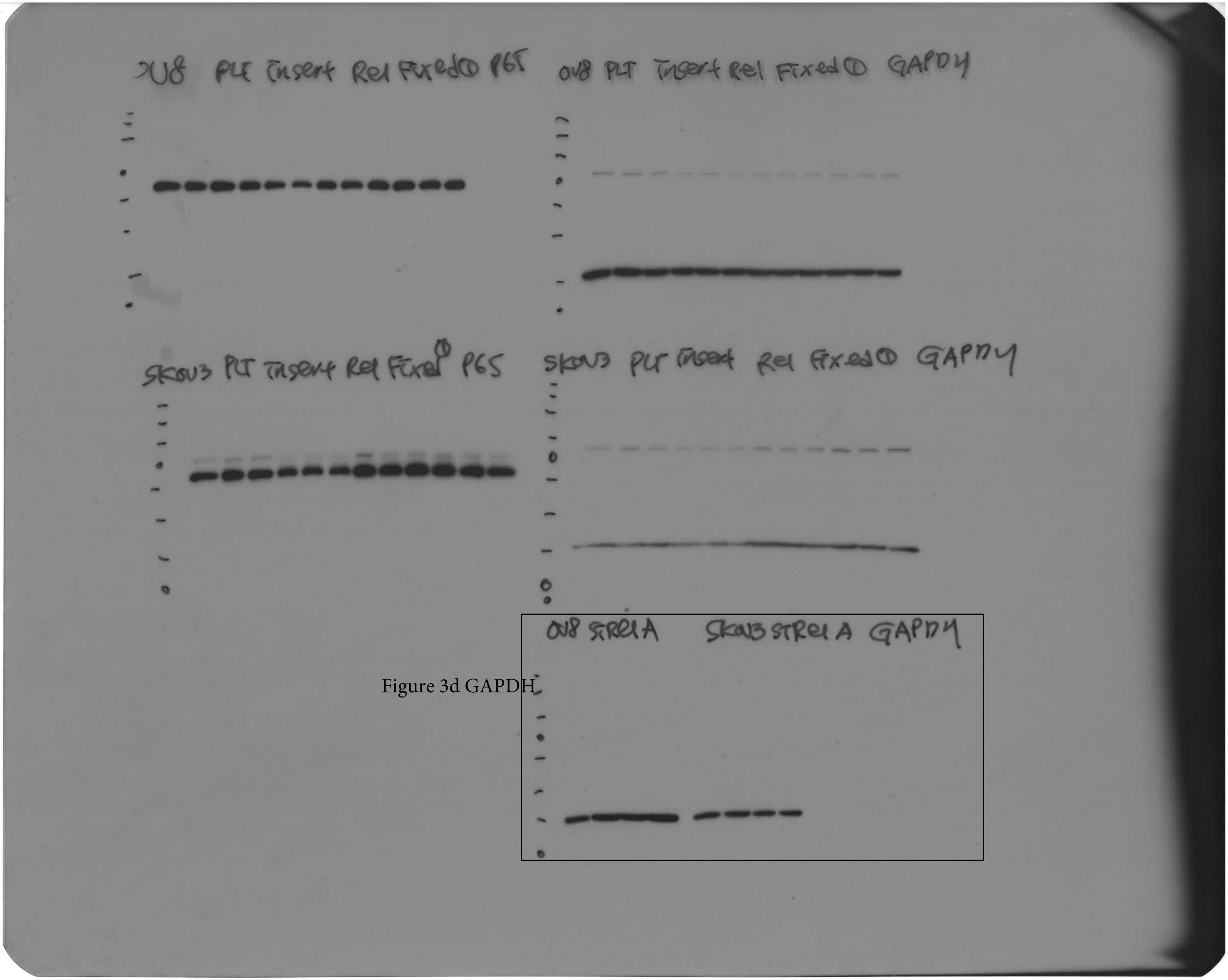

Figure 3d GAPDH

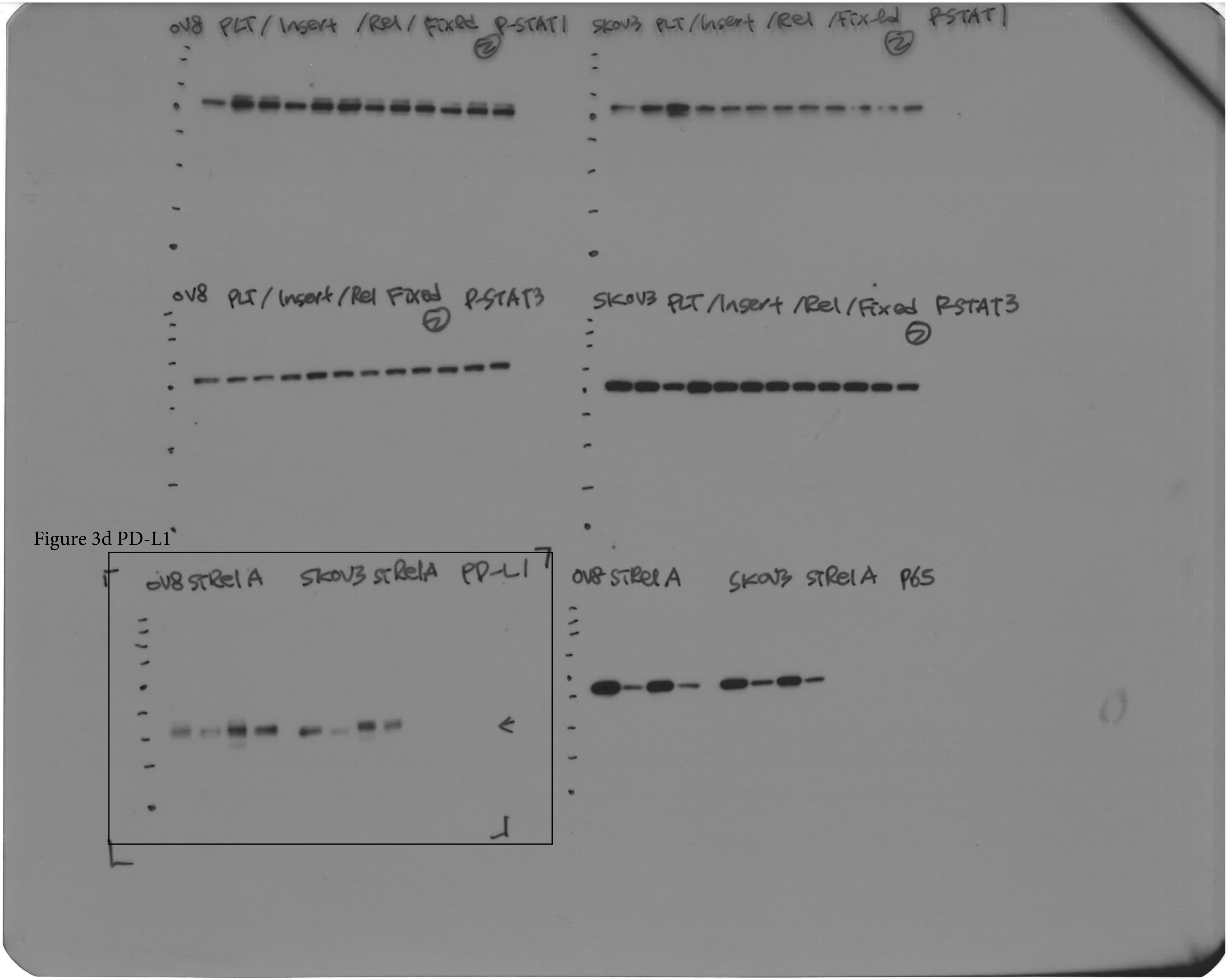

Figure 3d PD-L1

Figure 3d RelA

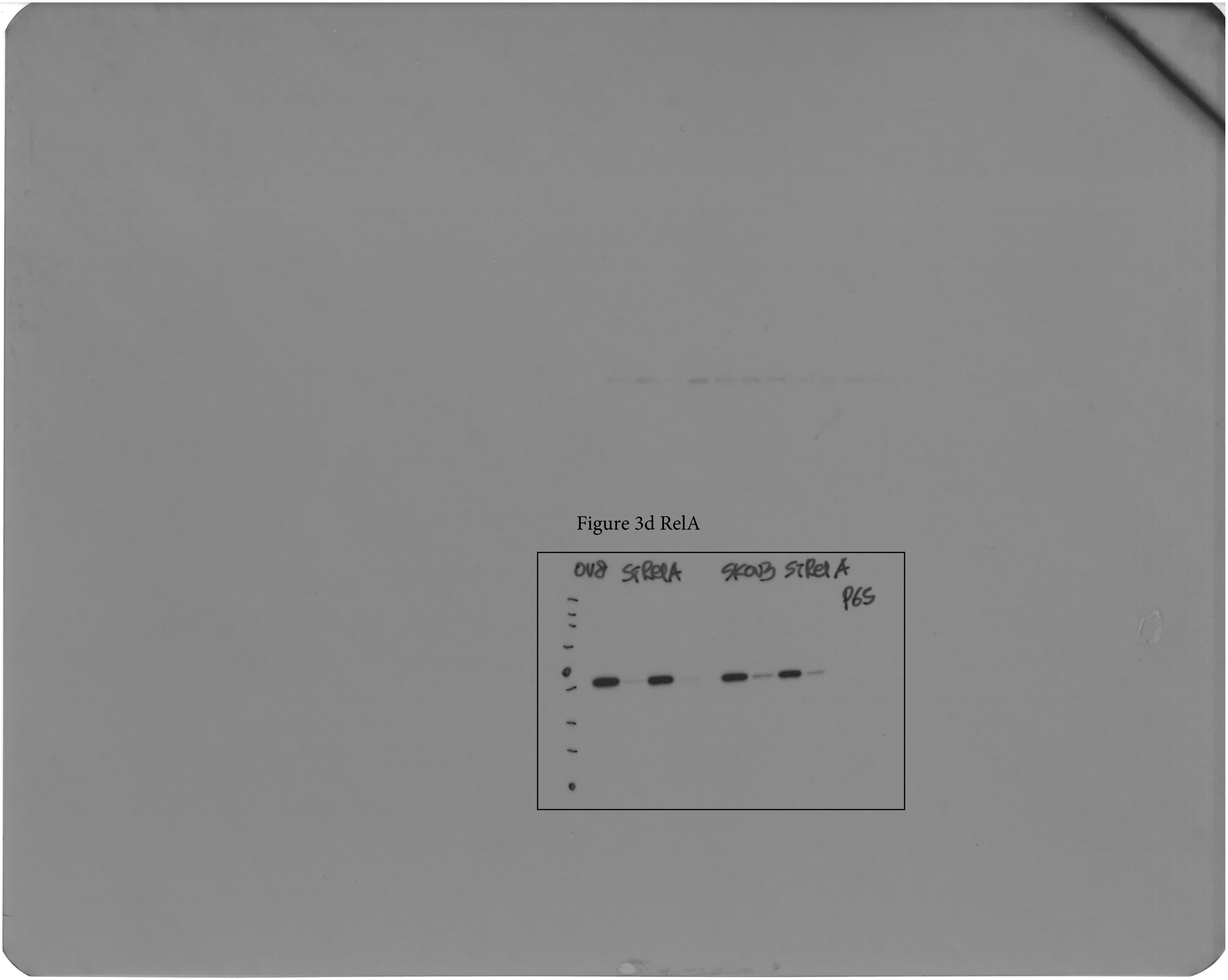

Figure 3e,f GAPDH

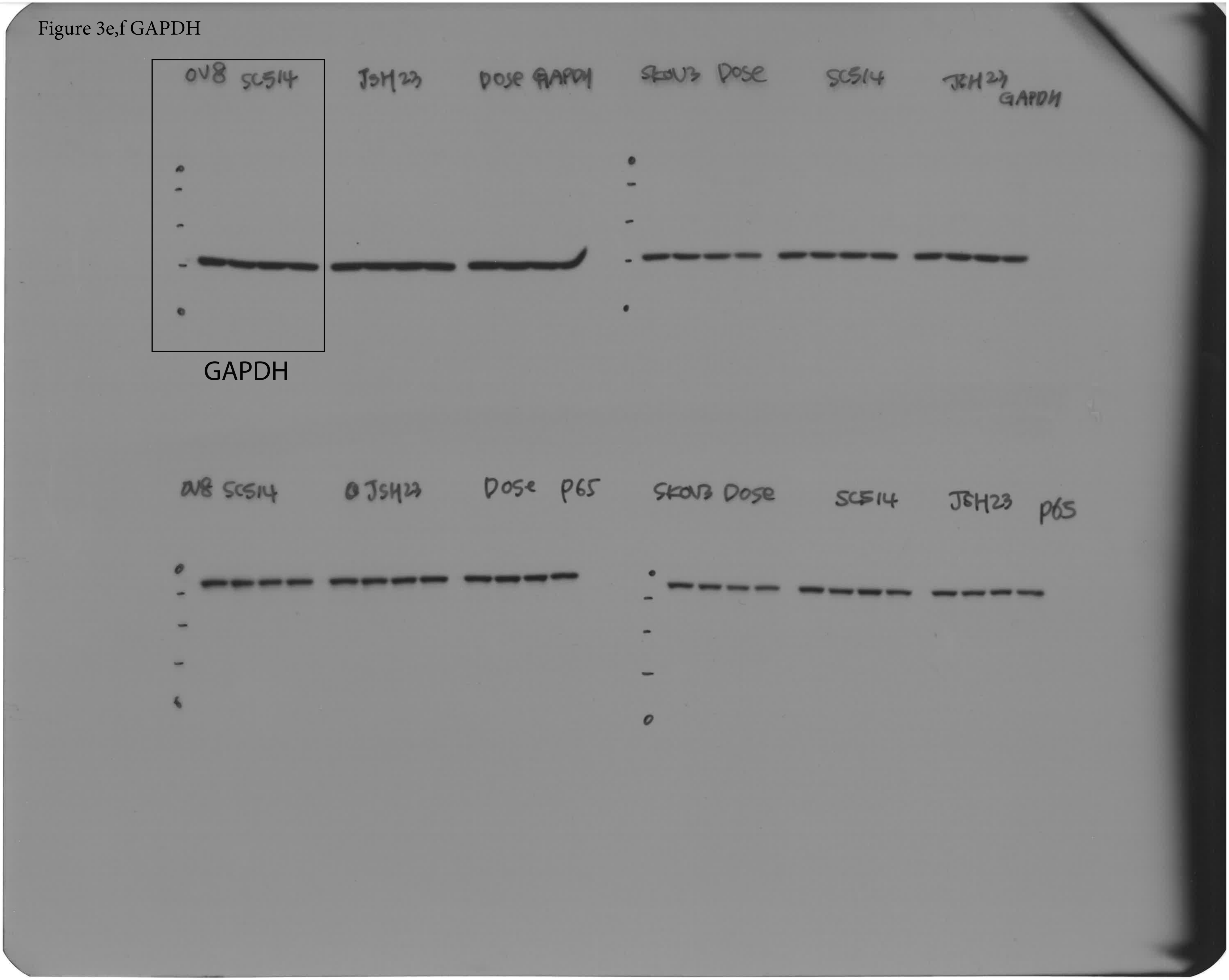

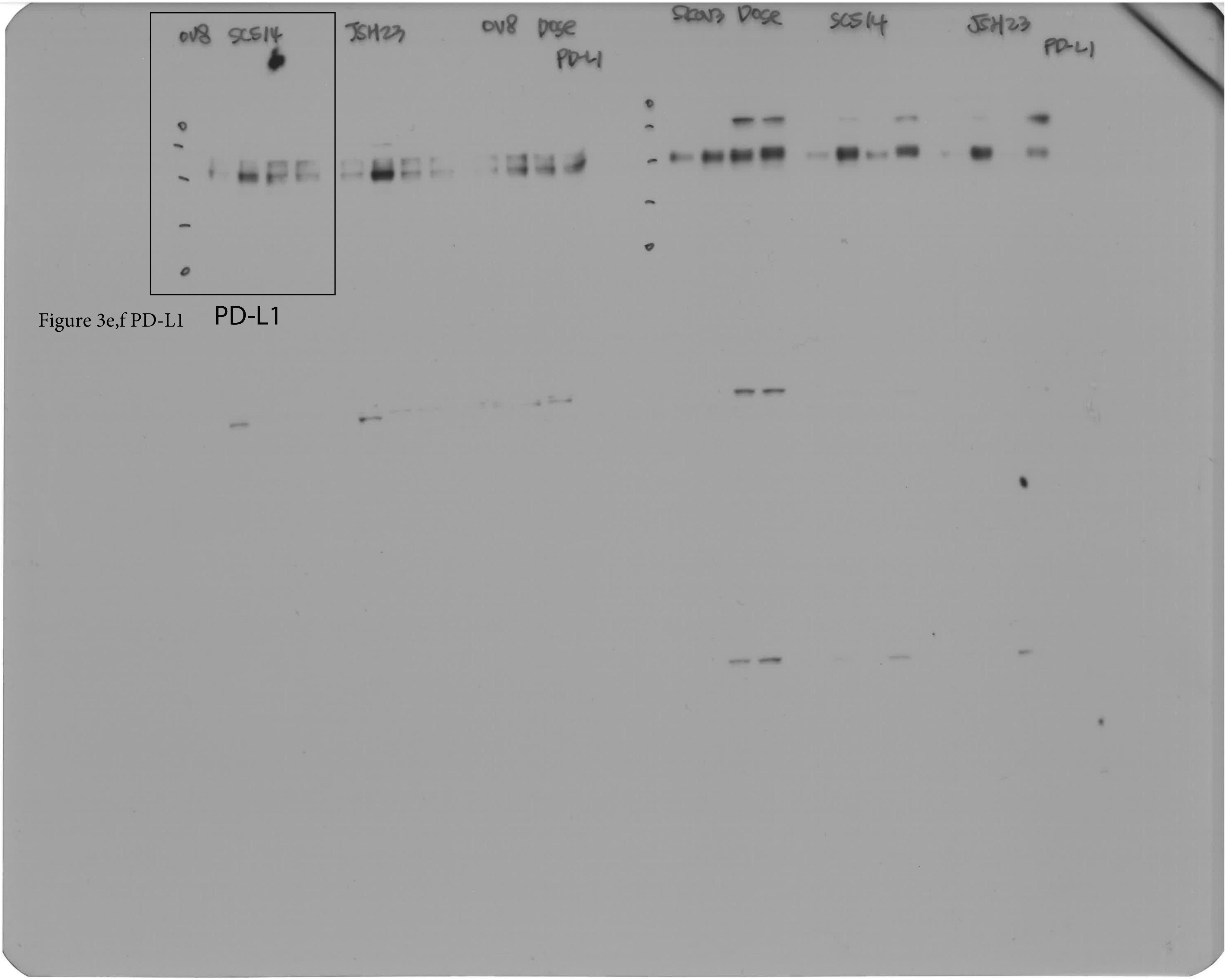

Figure 3e,f PD-L1

PD-L1

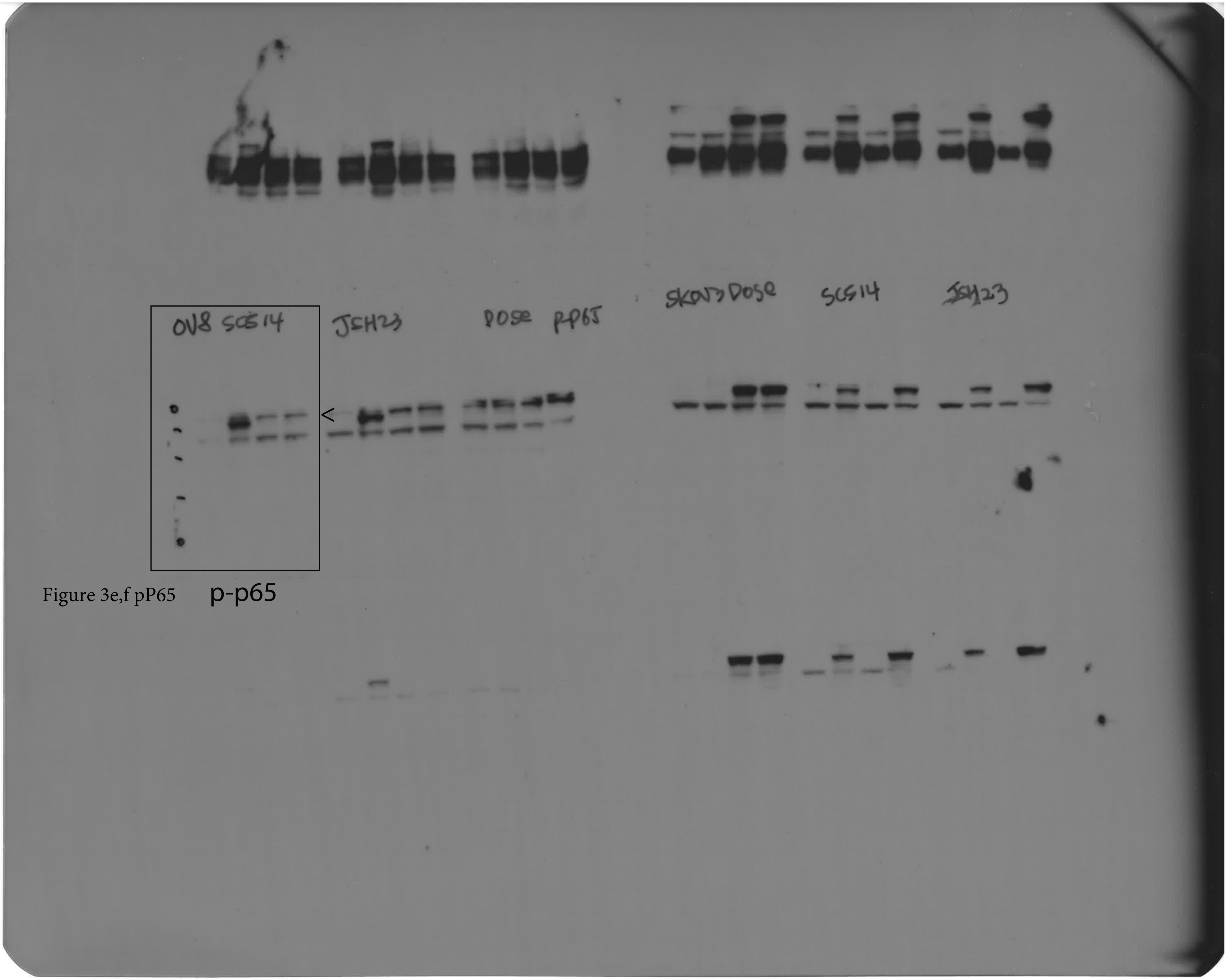

Figure 3e,f pP65

p-p65

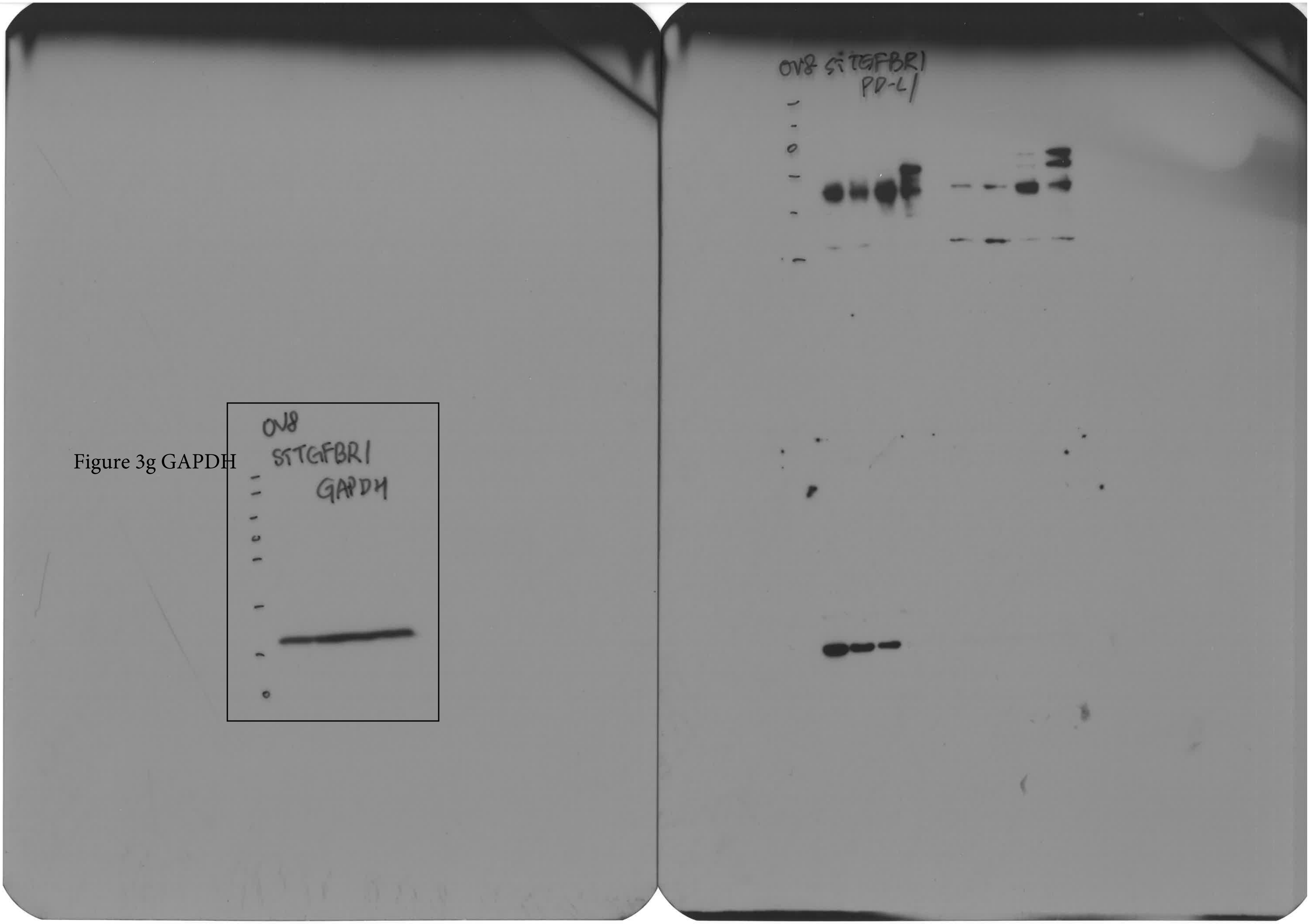

Figure 3g GAPDH

Figure 3g PD-L1

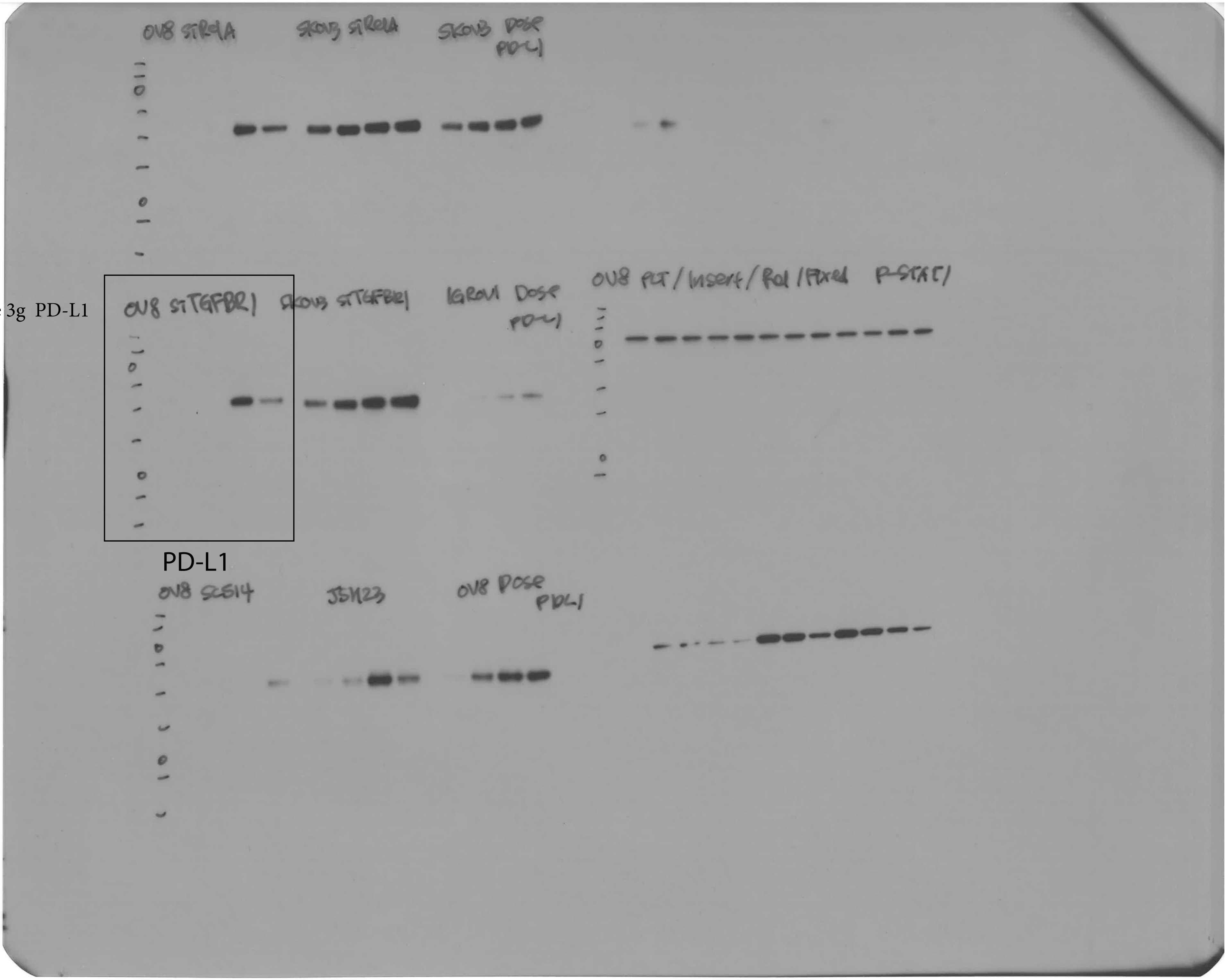

PD-L1

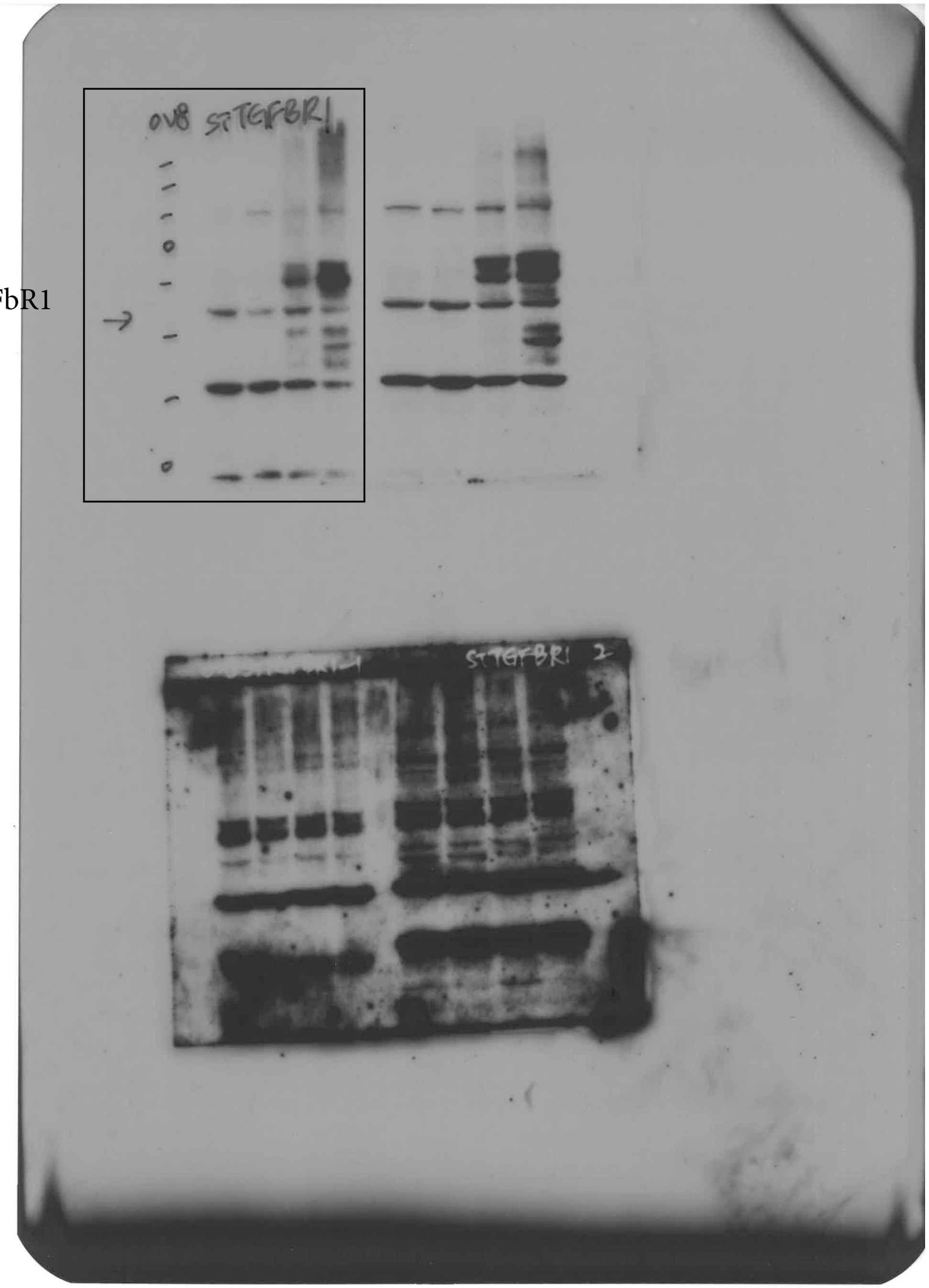

Figure 3g TGFbR1

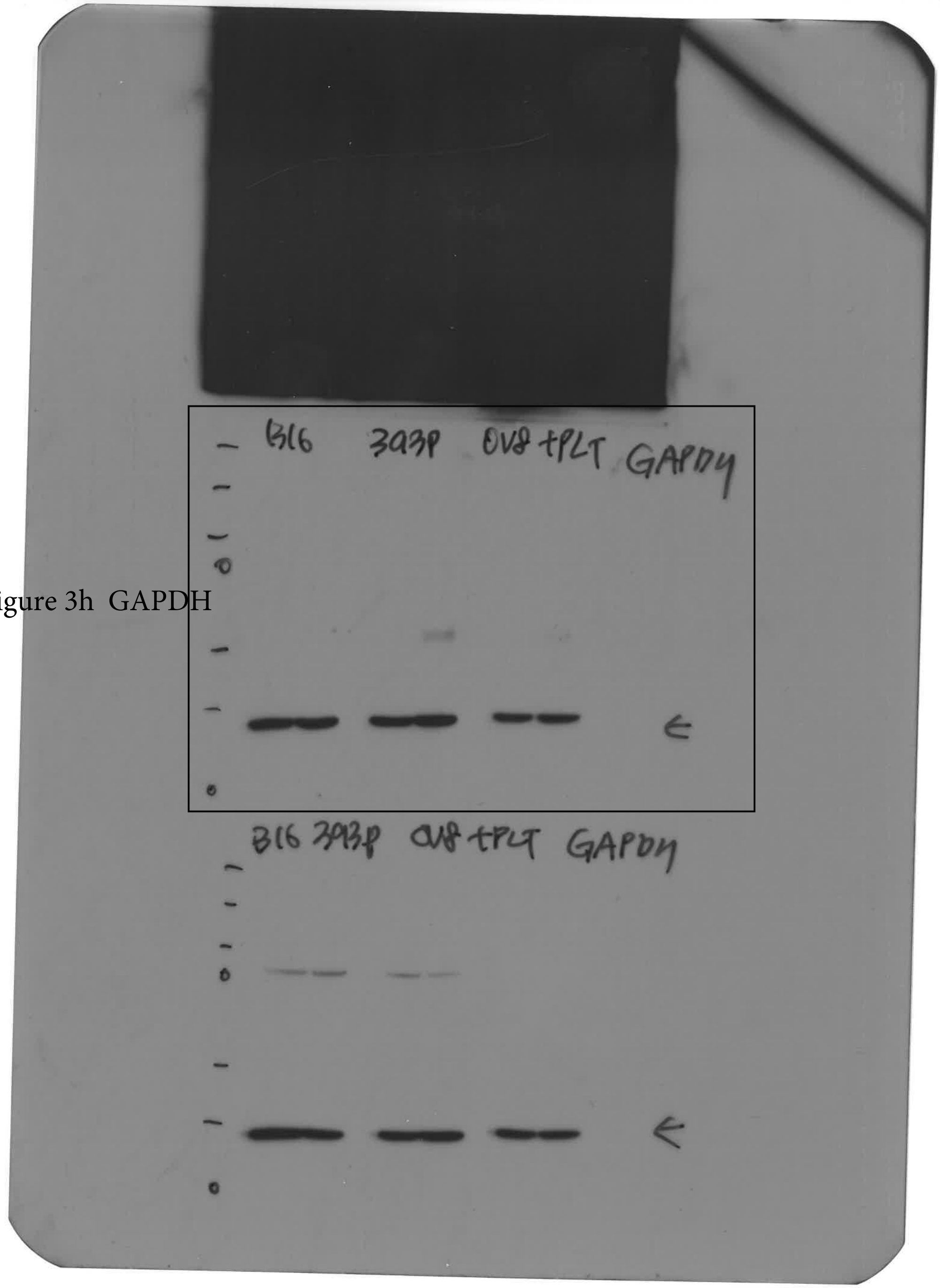

Figure 3h GAPDH

Figure 3h PD-L1

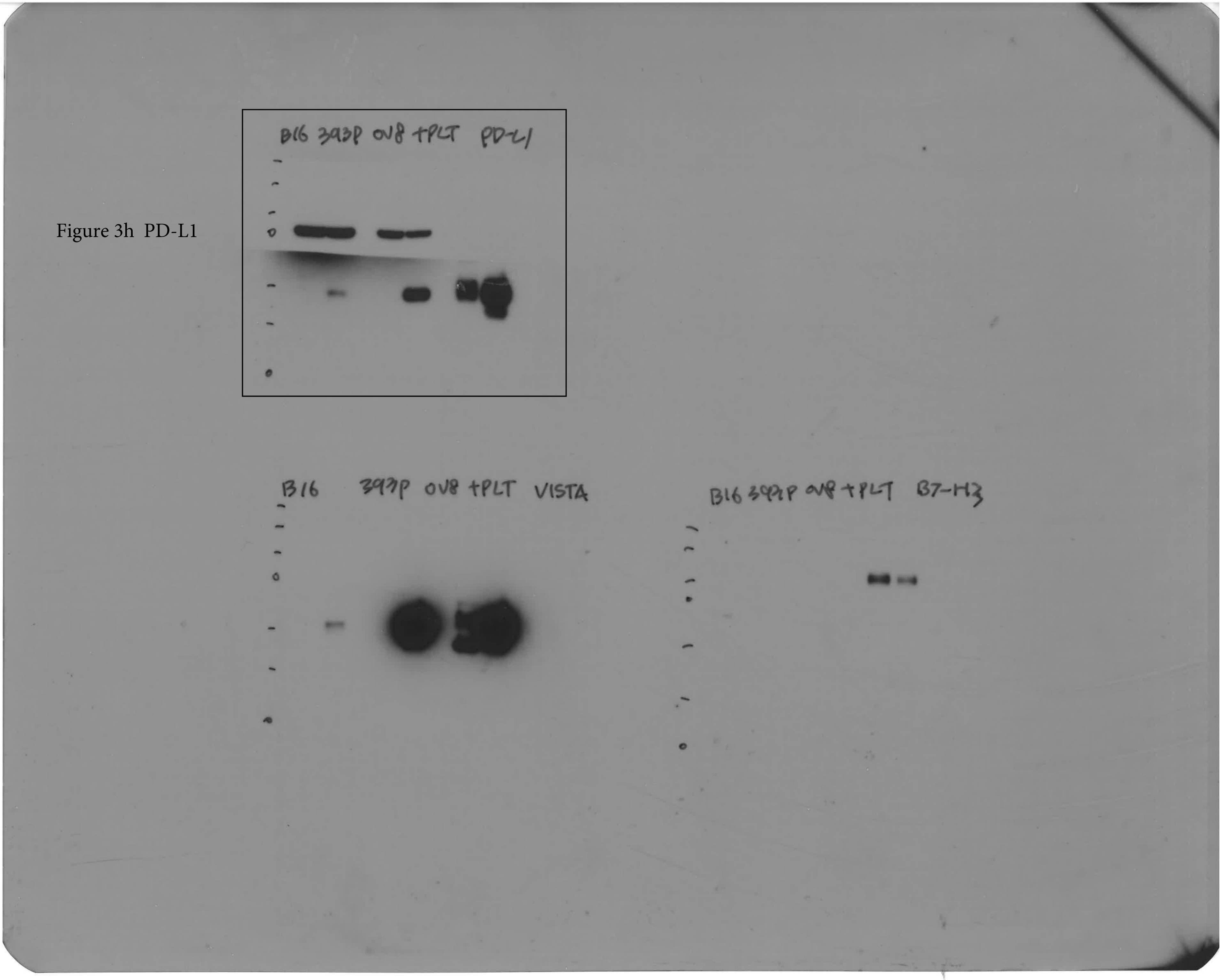

Figure 3i GAPDH

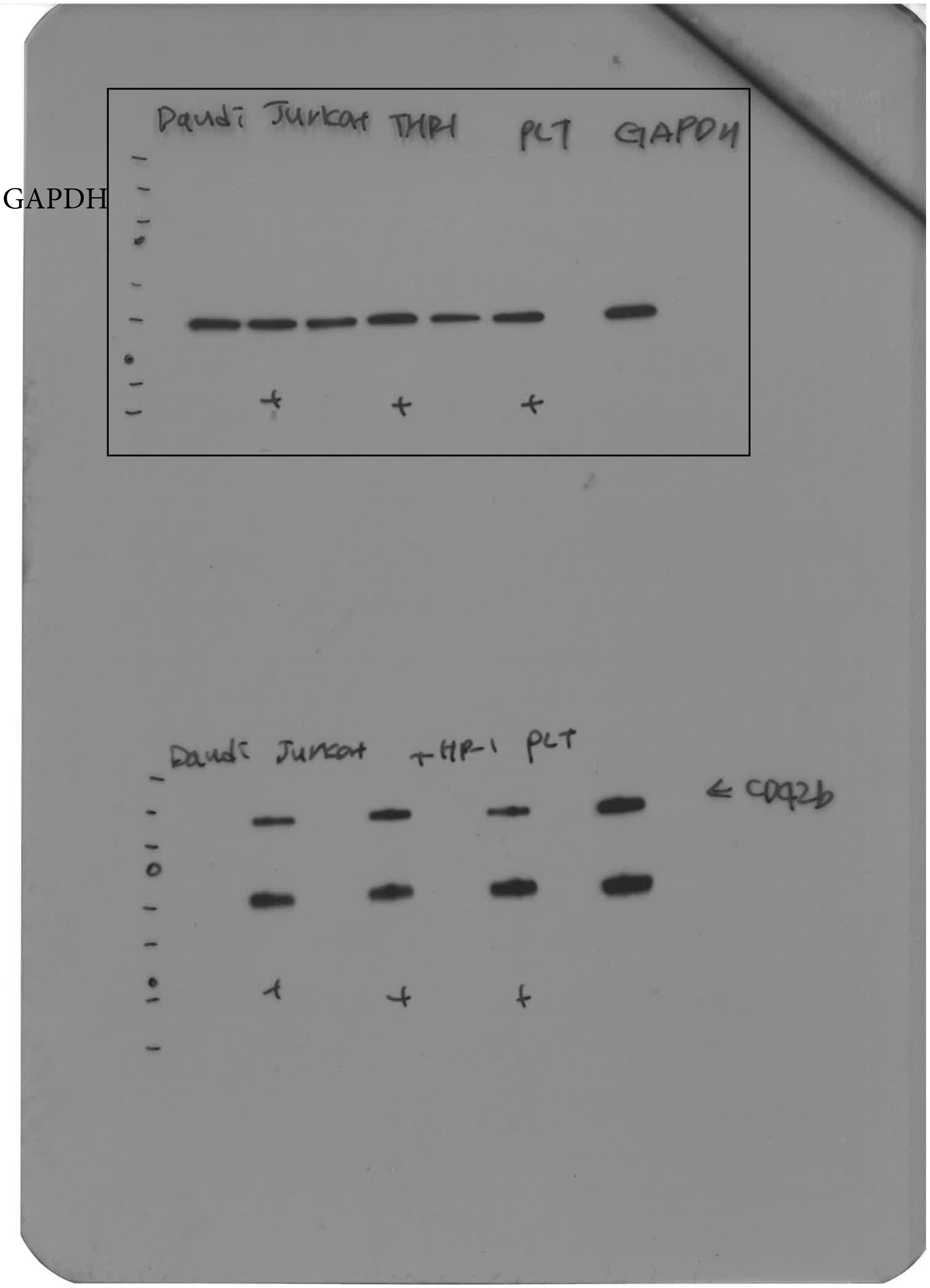

Figure 3i PD-L1

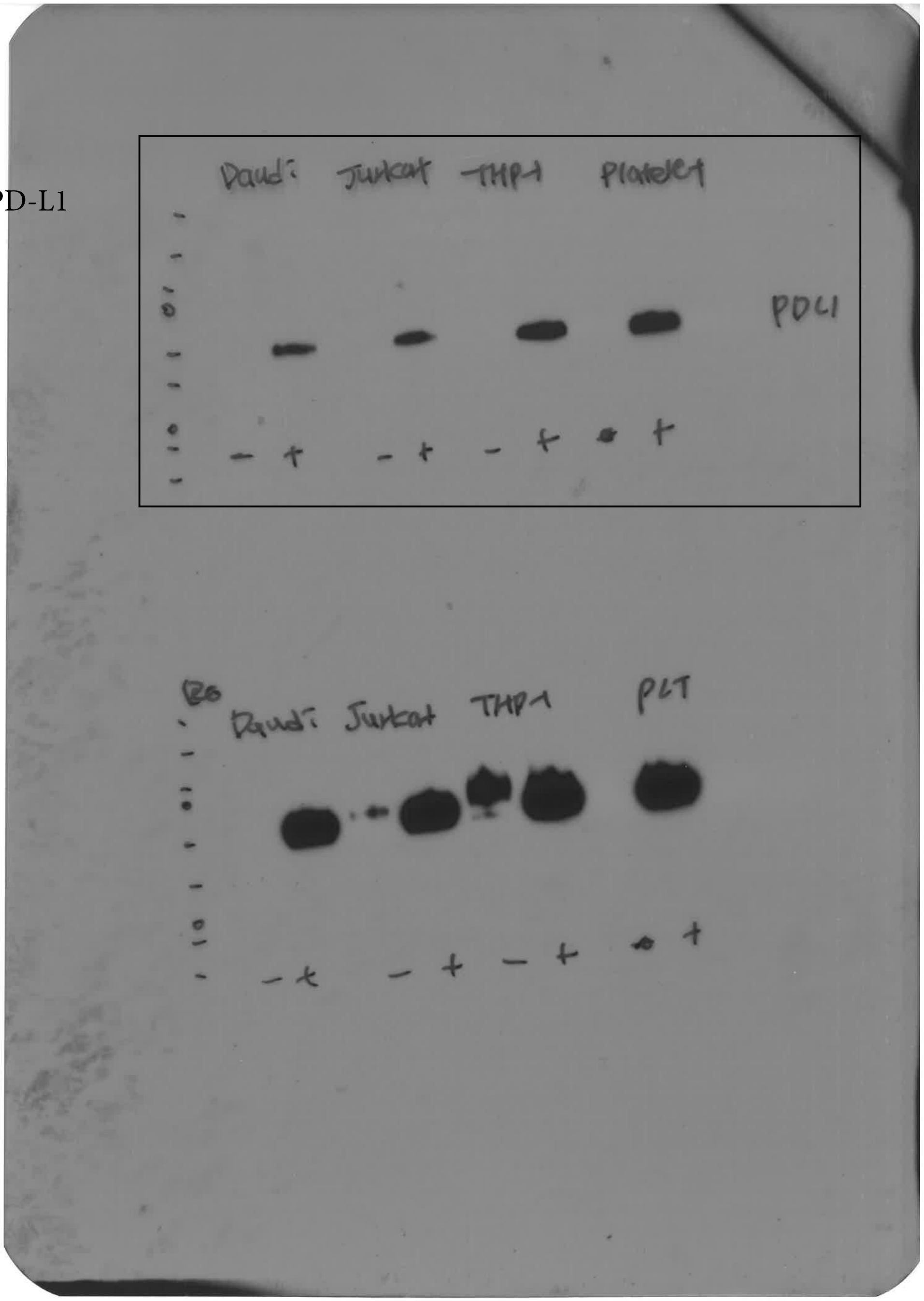

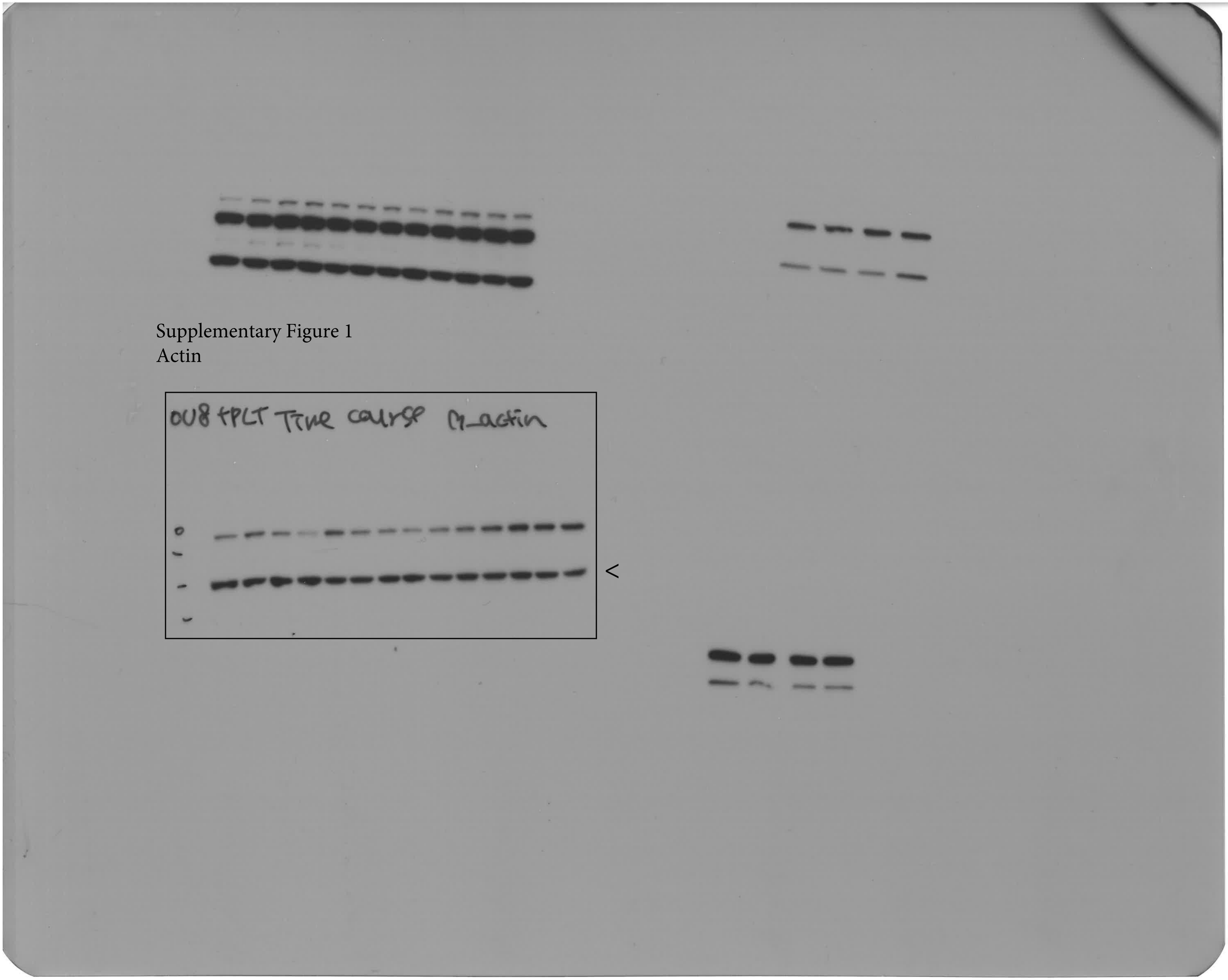

Supplementary Figure 1
Actin

Supplementary Figure 1
PD-L1

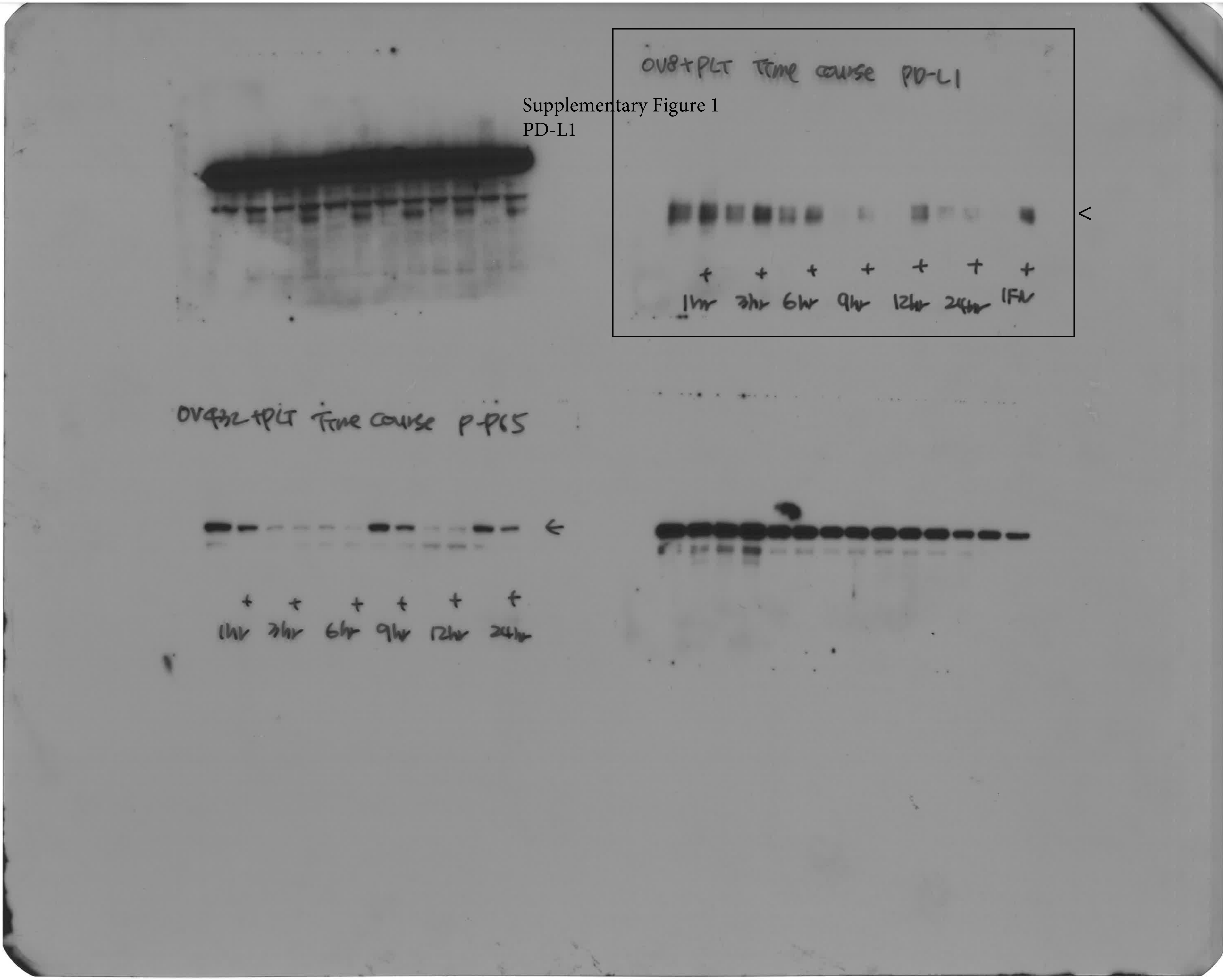

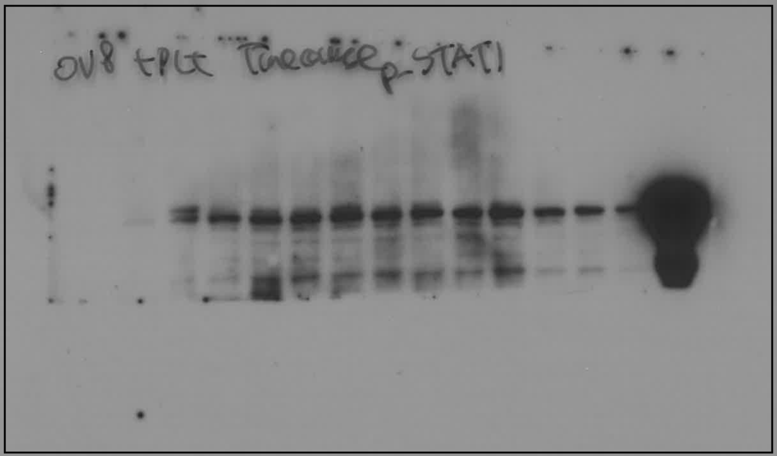

Supplementary Figure 1
p-STAT1

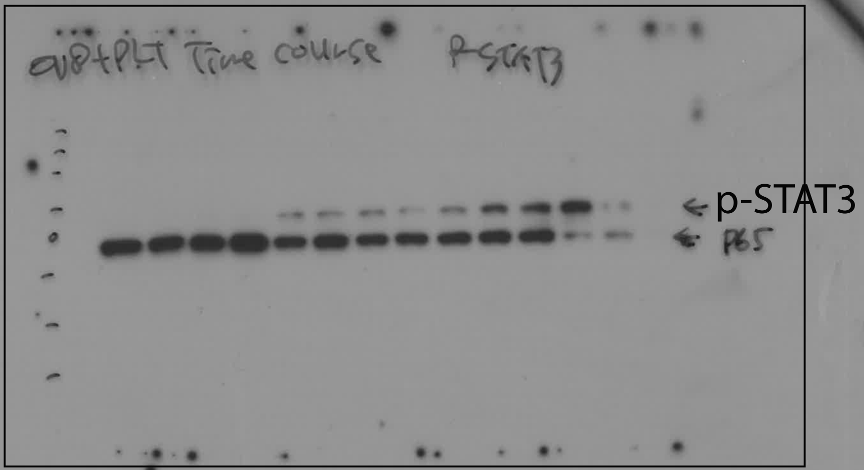

Supplementary Figure 1
p-STAT3

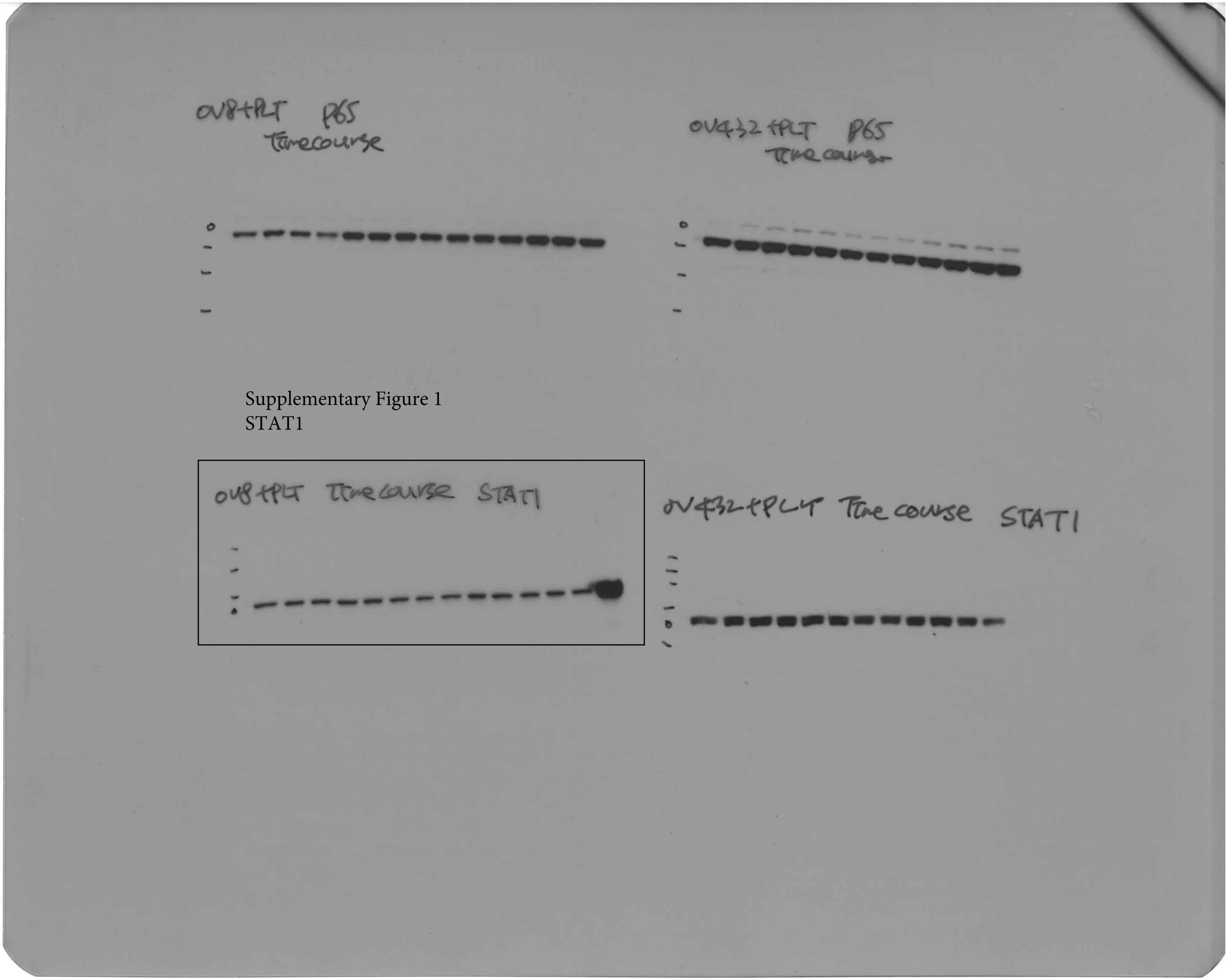

Supplementary Figure 1
STAT1

Supplementary Figure1
STAT3

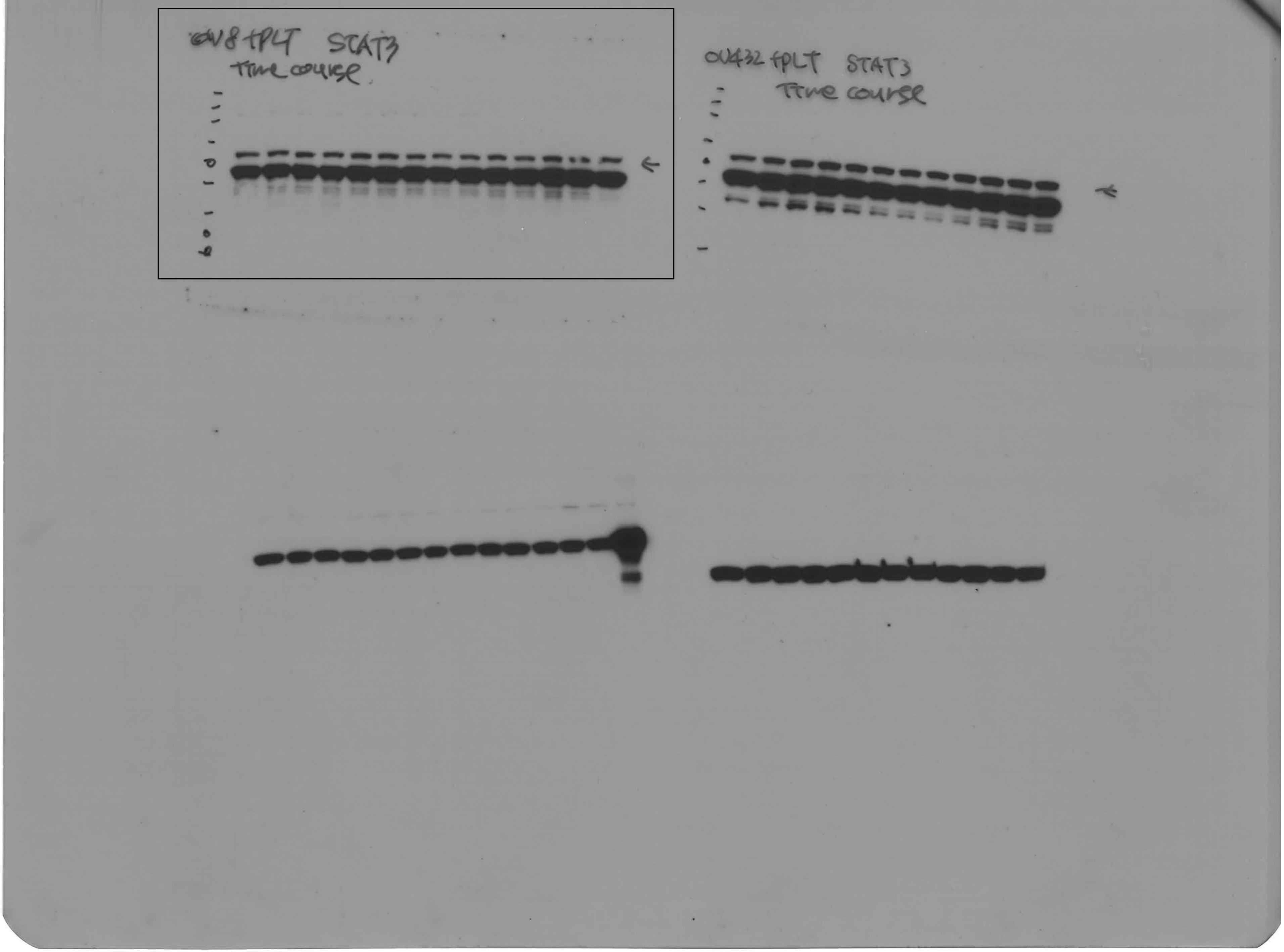

Supplement: Supplementary file 1 [file cancers-14-02498-s001.zip › Supplementary File S1.pdf]
